# Supplementary material for: Integrated Transcriptomics, Metabolomics, and Lipidomics Profiling in Rat Lung, Blood, and Serum for Assessment of Laser Printer-Emitted Nanoparticle Inhalation Exposure-Induced Disease Risks
Source: Int J Mol Sci. 2019 Dec 16;20(24):6348. doi: 10.3390/ijms20246348 (PMC6940784; doi:10.3390/ijms20246348)
Supplement: Supplementary file 1 [file ijms-20-06348-s001.pdf]

Supplementary Materials and Methods

Lipid analysis by LC-MS

Lipid profiling were performed on the ACQUITY UPLC coupled with a Xevo G2-XS QTOF/MS detector (Waters Corp. Milford, MA, USA) using ACQUITY BEH C18 column (Waters Corp.) (Dimension 100 × 2.1 mm, 1.7 µm particle size) maintained at 55°C. For lipid profiling, the columns contained mobile phase solvent A (2:1:1 v/v 5mM ammonium acetate in water: acetonitrile: isopropanol), 20µM phosphoric acid and 0.05% acetic acid) and B (9:1v/v 5mM ammonium acetate in isopropanol: acetonitrile, 0.05% acetic acid) for establishing the optimized gradient as follows: 0 min, 1% B; 2 min, 40% B; 11.5 min, 95% B; 12.0–12.50 min, 99.9% B; 12.55 min, 65% B; 12.65 min, 30% B; 12.75-14.25 min, 1% B[1]. Mass spectrometry analysis (QTOF/MS) was conducted in positive and negative ion modes as follows: capillary voltage, 2.0 kV/−1.5 kV; cone voltage 25; source temperature, 120°C; cone gas flow, 150 l/h; desolvation temperature and flow at 600°C and 1000 l/h; scan range, 50–2000 m/z; data acquisition rate, 0.1s and collision energy 4[1]. Data was acquired using Masslynx 4.1 workstation and UPLC-QTOF/MS Acquisition software (Waters Company, Milford, MA, USA) in centroid mode and m/z values were adjusted by LockSpray using leucine-enkephalin as lock mass compound for the positive ($[M + H]^+ = 556.2771$) and negative ($[M - H]^- = 554.2615$) ion modes respectively. The QC sample was injected 20 times before experiment to equilibriate the UPLC-MS system , further injection was done at the start, end and every 10th sample collection for reproducibility. A sequence of 24 diluted OC samples was injected during analysis run to detect “noise” in identified features. For targeted fatty acids LC-MS/MS analysis, the organic phase in ESI Negative form was employed. Elution gradient consist of mobile phase G (50:50 acetonitrile: water) and H (100% acetonitrile) was started with 65% mobile phase H, and linearly increased to 100% by 6 min and held for 3 min. Further 2 min equilibration was performed using initial conditions used for injection at 5 µl (flow rate 0.4 ml/min, 50°C). Statistical analysis was carried out using 2-way ANOVA followed by post-hoc multiple correction using FDR with Benjamini, Krieger and Yekutieli method [2].

Metabolite profiling

Supernatants were analyzed using the BEH HILIC column (1.7µm, 2.1x100 mm) on an ACQUITY UPLC coupled with a Xevo G2-XS QTOF/MS detector (Waters Corp. Milford, MA, USA) using mobile phase C (20 mM ammonium formate, 0.1% formic acid) and D (acetonitrile, 0.1% formic acid) gradient at 0 min, 95% B; 4.6 min, 80% B; 5.5 min, 50% B; 7.10-12.65 min, 95% B[1]. QTOF/MS acquisition parameters were as follows: capillary voltage, 1.5 kV; cone voltage 20; source temperature, 120°C; cone gas flow, 150 l/h; desolvation temperature 600°C and flow rate 1000 l/h; scan range, 50–2000 m/z; and collision energy 4.0. The QC samples were injected as described above under lipid profiling[1].

Metabolomics data processing and pathway analysis

Progenesis QI (V2.3) software was used for automatic data pre-processing, including retention time alignment, peak picking, deconvolution, and identification of features[3]. Retention time (RT)-mass/charge ratio (m/z) were used to characterize detected ions and placed into R (V3.4.2) for further data processing and statistical analysis. Locally estimated scatterplot smoothing (LOESS) regression was employed to correct for run order effects. Features were filtered according to the coefficient of variation (among QC injections) with correlation to dilution factors (among diluted QC injections). Principal component analysis (PCA) was employed to validate technical reproducibility of the analytical approach and visualize clustering, trends including outliers. Orthogonal partial least squares discriminant analysis (OPLS-DA) was next applied to screen differential variables responsible for differences between groups. Student's *t*-test was then used to further shortlist biomarker candidates based on FDR corrected *p*-values. Derived m/z values were matched to the putative metabolite using the online Metlin, HMDB and LipidMap databases and analyzed using MetaboAnalyst 4.0 pathway analysis[4]. Over Representation Analysis (ORA) was performed to assess for biologically meaningful patterns using the hypergeometric test and the adjusted one-tailed *p* values are provided after multiple testing. Pathway topology analysis was next performed using relative betweenness centrality to measure the number of shortest paths between node and global network topology. Disease enrichment pathway analysis was also carried out with disease-associated metabolite sets which consist of 344 metabolite sets reported in human blood, and we delineate pathway illustrating 2 or more compounds.

Supplementary Figures

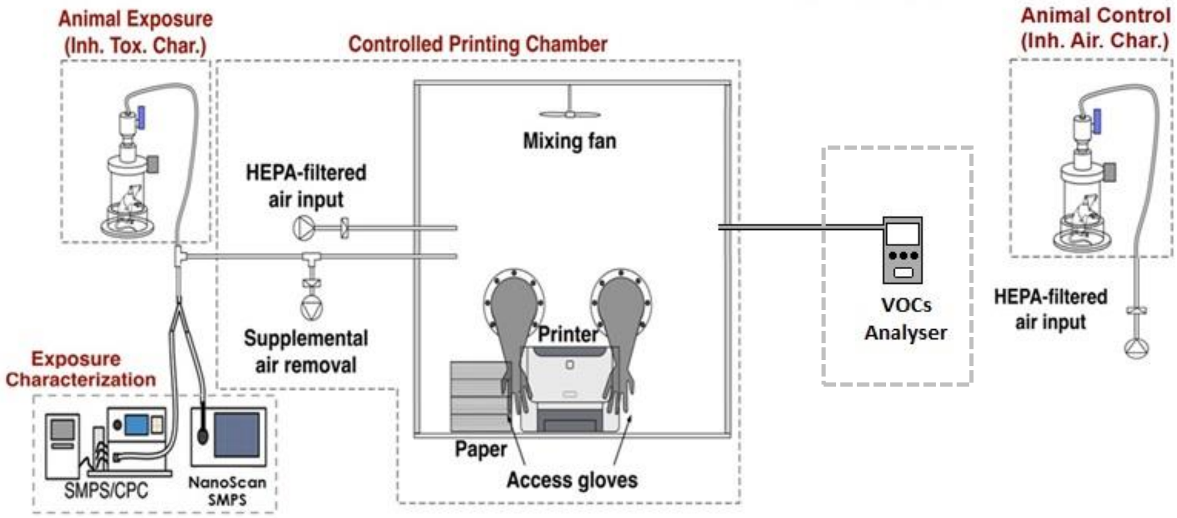

Figure S1. Printer Exposure Generation System (PEGS). Figure adapted from Pirela et al 2019 (NanoImpact).

Supplementary Tables

Table S1. Real-time particle characterization from the whole-body PEPs exposure for 1, 5, 9, 13, 17, or 21 days. CMD: count median diameter; GSD: geometric standard deviation. Table adapted from Pirela et al 2019 (NanoImpact).

Exposure Day	Particle density (g/cm^3)	CMD (nm)	GSD	Mass concentration ($\mu g/m^3$)
1	1.2	43.76	1.65	48.10
5	1.2	44.78	1.66	60.31
9	1.2	44.13	1.68	61.60
13	1.2	44.64	1.70	58.96
17	1.2	45.75	1.70	64.41
21	1.2	44.00	1.71	76.43

Table S2. Top significant (p<0.05) biological processes, molecular functions, and diseases in PEPs-exposed rat lung tissue in gene ontology analysis. Up-regulated genes are highlighted in red, and down-regulated genes are highlighted in blue. Genes with a statistically significant (p<0.05) differential expression are highlighted in bold.

Post-exposure time points	Biological processes	Molecular functions	Diseases
Day 1	epoxygenase P450 pathway (***Cyp2c22, Cyp2b3, Cyp2a1***, ***Cyp2c6v1,*** *Cyp2c11*, *Cyp2f4, Cyp2c13, Cyp2a3*)	steroid hydroxylase activity (***Cyp8b1, Cyp2c22, Cyp3a18, Cyp2b3, Cyp2a1, Cyp2c6v1, Cyp3a23/3a1,*** *Cyp1a1, Cyp2c11,Cyp2f4, Cyp2c13, Cyp2a3*)	Inherited thrombophilia (***F2***, ***Fgb***, ***Serpinc1,*** *Fga, Plg*)
	negative regulation of endopeptidase activity (***LOC299277***; ***Serpinf2***, ***Ahsg, Pzp, Serpinc1***, ***Itih1***, ***Mug1***, ***Itih2, Itih3***, ***Itih4***, ***Ambp***, ***Mug2,*** *Hrg, LOC297568, Fabp1, Naip6, Serpina3c*)	heme binding (***Nr1d1***, ***Cyp4a3***, ***Cyp8b1***, ***Cyp2c22***, ***Cyp3a18***, ***Cyp2b3***, ***Cyp2a1, Ambp, Cyp2c6v1, Cyp3a23/3a1,*** *Hrg, Cyp1a1, Cyp2c11, Cyp2f4, Cyp2c13, Mb, Cyp2a3*)	Familial amyloidosis (***Ttr,*** *Apoa2, Fga*)
	negative regulation of fibrinolysis (***Cpb2, Serpinf2***, ***Apoh,*** *Hrg, Plg*)	serine-type endopeptidase inhibitor activity (***Serpinf2***, ***Pzp***, ***Serpinc1***, ***Itih1***, ***Mug1***, ***Itih2***, ***Itih3***, ***Itih4***, ***Ambp***, ***Mug2,*** *Hrg, LOC297568, Serpina3c;* ***LOC299277***)	Tyrosinemia (***Tat,*** *Hpd*)
	acute-phase response (***F2***, ***Ahsg***, ***Saa4***, ***Mug1***, ***Itih4***, ***Crp,*** *Fga, Hamp*)	oxidoreductase activity, acting on paired donors, with incorporation or	Familial advanced sleep phase syndrome (***Per3***, ***Per2***)

		reduction of molecular oxygen, reduced flavin or flavoprotein as one donor, and incorporation of one atom of oxygen (***Cyp2c22***, ***Cyp3a18***, ***Cyp2b3***, ***Cyp2a1***, ***Cyp2c6v1, Cyp3a23/3a1,*** *Cyp1a1, Cyp2c11, Cyp2f4, Cyp2c13, Cyp2a3*)	
	circadian regulation of gene expression (***Nr1d1***, ***Bh1he40***, ***Per3***, ***Ciart***, ***Per2***; ***Arntl***)	arachidonic acid monooxygenase activity (***Cyp4a3***, ***Cyp2c22***, ***Cyp2b3, Cyp2a1***, ***Cyp2c6v1***)	Afibrinogenemia; Dysfibrinogenemia (***Fgb,*** *Fga*)
Day 5	myofibril assembly (*Myoz2, Mybpc3, Myom2, Tnnt2, Actn2, Csrp3*)	actin binding (*Myoz2, Tnnc1, Mybpc3, Tnni3, Myom2, Tnnt2, Actn2, Csrp3*)	Dilated cardiomyopathy (***RT1-Bb,*** *Tnnc1, Mybpc3, Tnni3, Tnnt2, Actn2, Csrp3*)
	ventricular cardiac muscle tissue morphogenesis (*Tnnc1*, *Mybpc3*, *Tnni3, Tnnt2*)	MHC class I protein binding (***RGD1566006***, *LOC100910669;* ***LOC680910***)	Hypertrophic cardiomyopathy (*Tnnc1, Mybpc3, Tnni3, Tnnt2, Csrp3*)
	regulation of muscle filament sliding speed (*Tnnc1, Tnnt2*)	troponin C binding (*Tnni3, Tnnt2*)	Restrictive cardiomyopathy (*Tnni3, Tnnt2*)
	striated muscle contraction (*Smpx, Tnnc1, Mybpc3, Mb, Tnni3, Myom2, Tnnt2, Csrp3*)	FATZ binding (*Myoz2, Actn2*)	Skeletal defects, genital hypoplasia, and mental retardation (***Zbtb16***)

	negative regulation of ATPase activity (*Tnni3, Tnnt2)*	structural constituent of muscle (*Mybpc3, Myom2, Csrp3*)	Nephrogenic syndrome of inappropriate antidiuresis (***Avpr2***)
Day 9	complement activation, classical pathway (*Igh-6, C5; Ighg1*)	immunoglobulin receptor binding (*Ighg1; Igh-6*)	Familial idiopathic ventricular fibrillation (***Scn5a***)
	regulation of stem cell division (***Esrrb***, ***Sfrp2***)	antigen binding (***LOC362795,*** *Ighg1*; Igh-6)	Progressive cardiac conduction defect; Progressive familial heart block; Lenegre-Lev disease (***Scn5a***)
	defense response to bacterium (*Naip6, Ighg1, Defb4, Bpifa1; Igh-6, Lpo*)	manganese ion binding (***Ppef2; Idi1***)	Kearns-Sayre Syndrome (*ND3*)
	response to light intensity (***Hmgcs1***; *ND3*)	thiocyanate peroxidase activity (*Lpo*)	Leber hereditary optic neuropathy and dystonia (*ND3*)
	positive regulation of peptidyl-serine phosphorylation (***Oprd1; Stox1, Sfrp2***)	enkephalin receptor activity (***Oprd1***)	Dilated cardiomyopathy (***Scn5a***, *Tnnc1*)
Day 21	axoneme assembly (***Rsph4a***, ***Dnai1***, ***Pih1d3***, ***Iqcg***, ***Spag16***, ***Cfap206***, ***Armc4***, ***Dnah5***, ***Spef2***, ***Dnah12***, ***Ccdc39***, ***Dnai2***, ***Ak7, Ccdc151***, ***Dnah7***, ***Lrrc6***, ***Rp1***, ***Spag17***, ***Rsph1,*** *Drc1*)	dynein light chain binding (***Dnai1***, ***Dnah11***, ***Dnah5***, ***Dnah12***, ***Dnai2***, ***Dnah7***, ***Dnah6***, *Dnah9*)	Primary ciliary dyskinesia (***Rsph4a***, ***Dnai1***, ***Dnah14***, ***Dnah11***, ***Dnah5***, ***Dnah12***, ***Dnai2***, ***Dnah7***, ***Lrrc6***, ***Dnah3***, ***Dnah6***, ***Rsph1***, *Drc1*, *Dnah9*)

	cilium movement (***Nme5***, ***Tekt1***, ***Rsph4a***, ***Dnai1***, ***Pih1d3***, ***Spag16***, ***Ropn1l***, ***Cfap206***, ***Armc4***, ***Dnah11***, ***Dnah5, Spef2***, ***Dnah12***, ***Ccdc39***, ***Dnai2***, ***Ak7***, ***Ccdc151***, ***Dnah7***, ***Lrrc6***, ***Spag17***, *Dnah9*)	dynein intermediate chain binding (***Dnah11***, ***Dnah5***, ***Dnah12***, ***Dnah7***, ***Dynlrb2***, ***Dnah6***, *Dnah9*)	Hypertrophic cardiomyopathy (***Tnnc1***, *Actc1, Tnni3, Tnnt2, Csrp3, Ttn, Mybpc3, Myh6*)
	myofibril assembly (*Actc1, Tnnt2, Myom2, Mybphl, Csrp3, Myoz2, Casq2, Actn2, Ttn, Lmod2, Mybpc3, Myh6*)	ATP-dependent microtubule motor activity, minus-end-directed (*Dnah11, Dnah5, Dnah12, Dnah7, Dnah6, Dnah9*)	Dilated cardiomyopathy (***Tnnc1***, *Actc1, Tnni3, Tnnt2, Csrp3, Actn2, Ttn, Mybpc3 Myh6*)
	cardiac muscle tissue morphogenesis (***Tnnc1***, *Actc1, Tnni3, Tnnt2, Ryr2, Ttn, Mybpc3, Tbx20, Myh6*)	dynein light intermediate chain binding (***Dnah11, Dnah5, Dnah12, Dnah7, Dnah6***, *Dnah9*)	Restrictive cardiomyopathy (*Tnnt2, Tnni3, Actc1*)
	determination of left/right symmetry (***Dnai1***, ***Armc4***, ***Dnah11***, ***Dnah5***, ***Ccdc39***, ***Dnai2***, ***Ccdc151***, ***Lrrc6***, ***Daw1***, *Drc1, T, Tbx20*)	steroid hydroxylase activity (***Cyp39a1, Cyp2e1***, *Cyp1a1*; ***Cyp2d3***, ***LOC100361547***, *Cyp2g1, Cyp2f4, Cyp2a3*)	Left ventricular noncompaction (***Myh6***, *Tnnt2, Actc1*)

Table S3. Top significant (*p*<0.05) biological processes, molecular functions, and diseases in PEPs-exposed rat blood in gene ontology analysis. Up-regulated genes are highlighted in red, and down-regulated genes are highlighted in blue. Genes with a statistically significant (*p*<0.05) differential expression are highlighted in bold.

**Post-exposure time point**	**Biological processes**	**Molecular functions**	**Diseases**
Day 1	platelet activation (***Pear1, Gp5, Ppbp, Gp1ba, Itga2b, Vwf, Alox12, P2rx1, Treml1, P2ry12, Cd9, Itgb3, Vcl; F2r***)	MHC class I protein binding (***Cd244, RGD1559588****, RGD1561143; Pdia3*)	Macrothrombocytopenia (***Gp1bb, Tubb2a, Gp9, Gp1ba, Itga2b, Vwf, Itbg3, Actn1***)
	platelet formation (***Ppbp, Ptpn11, Tal1, Mpig6b, Actn1***)	ATPase activity, coupled to transmembrane movement of ions, rotational mechanism (***Atp6v0e1,*** *Atp6ap1l;* ***Atp5g1, Atp6v1f***)	Bernard-Soulier syndrome; Giant platelet syndrome (***Gp1bb, Gp9, Gp1ba***)
	integrin-mediated signaling pathway (***Ptpn11, Itga2b, Mpig6b, Loxl3, Itgb3, Plp1****, Itga6;* ***Nme2****, Cd177*)	thrombin-activated receptor activity (***F2r; F2rl2***)	Glanzmann thrombasthenia (***Itga2b****,* ***Itgb3***)
	establishment of synaptic specificity at neuromuscular junction (***F2r****,* ***Gphn***)	protease binding (***Vwf, Serpinb6a, Timp3, Itgb3****, Prkn,* ***Polg, Bfar, Lcn2****, Cd177*)	Hemophilia (***Gp1ba, Vwf***)
	ATP hydrolysis coupled proton transport	integrin binding	Scapuloperoneal myopathy (***Myh7****,* ***Fhl1***)

	(***Atp5g1, Atp6v1f, Atp6v0e1***, *Atp6ap1l*)	(***Cd151***, ***Vwf*, *Cd9, Itgb3, Actn1,*** *Itga6, Gpnmb, Cd177*)	
Day 5	negative regulation of endoplasmic reticulum unfolded protein response (***Wfs1, Bfar***, *Nck2*)	olfactory receptor activity (***Olr1422, Olr748, Olr480, Olr828, Olr1410, Olr130, Olr252, Olr1369, Olr448, Olr611, Olr1401, Olr569, Olr199, Olr832, Olr1471, Olr140, Olr1230, Olr653, Olr808, Olr522, Olr954, Olr119, Olr826, Olr499, Olr230, Olr168***, *Olr1539, Olr1192, Olr1332, Olr255, Olr1619*, ***Olr1587, Olr240***, *Olr339, Olr460, Olr1196, Olr1736, Olr67, Olr1537, Olr1569*)	Allergic rhinitis (***Xcr1, Ccr1***, *Ccr1l1*)
	G-protein coupled receptor signaling pathway (***Olr1422, Olr748, Olr480, Olr828, Olr1410, Olr130, Olr252, Olr1369, Olr448, Olr611, Htr1b, Rgs6, Olr1401, Olr569, Olr199, Olr832, Olr1471, Tyro3, Olr140, Glra1, Olr1230, Olr653, Olr808, Olr522, Olr954, Vom1r4, Rxfp1, Olr119, Olr826, Olr499, Olr230,***	chemokine receptor activity (***Xcr1, Ccr1, Cx3cr1***, *Ccr1l1*)	Meningioma (***Klf4, Smarce1l***)

	Olr168, *Olr1539*, *Olr1192*, *Ramp3*, *Olr1332*, *Ppbp*, *Olr255*, *Olr1619*, ***Olr1587***, ***Plcb1***, ***P2ry2***, ***Slc26a6***, ***Rgs4***, ***Gngt2***, ***Xcr1***, ***Gng13***, ***Olr240***, ***Qrfpr***, ***Ccr1***, ***Mrgprf***, ***Taar7d***, ***Olr339***, ***Vom1r89***, ***Cx3cr1***, *Olr1460*, *Olr1196*, *Olr1736*, *Vom1r92*, *Ccr1l1*, *Olr1537*, *Olr1569*)		
	detection of chemical stimulus involved in sensory perception of smell (***Olr1422***, ***Olr748***, ***Olr480***, ***Olr828***, ***Olr1410***, ***Olr130***, ***Olr252***, ***Olr1369***, ***Olr448***, ***Olr611***, ***Olr1401***, ***Olr569***, ***Olr199***, ***Olr832***, ***Olr1471***, ***Olr140***, ***Olr1230***, ***Olr653***, ***Olr808***, ***Olr522***, ***Olr954***, ***Olr119***, ***Olr826***, ***Olr499***, ***Olr230***, ***Olr168***, *Olr1539*, *Olr1192*, *Olr1332*, *Olr255*, *Olr1619*, ***Olr1587***, ***Olr240***, ***Olr339***, *Olr1460*, *Olr1196*,	tumor necrosis factor receptor binding (***Tnfsf13***, ***Erap1***, ***Tnfsf10***, ***Nucb2***)	Familial Mediterranean fever; Familial hereditary periodic fever syndromes (***Mefv***)

	Olr1736, Olr67, Olr1537, Olr1569)		
	regulation of muscle hyperplasia (***Klf4, Aif1***)	sodium-independent organic anion transmembrane transporter activity (***Slco3a1***, ***LOC685081***, ***Slco5a1***)	Fibrodysplasia ossificans progressive (***Acvr1***)
	response to platinum ion (***Alad***, *Alb*)	acetyl-CoA C-acyltransferase activity (***Acaa1a, Scp2***)	Wolfram syndrome; DIDMOAD syndrome (***Wfs1***)
Day 9	detection of chemical stimulus involved in sensory perception of smell (***Olr1621, LOC679803, Olr1692, Olr1472, Olr551, Olr894, Olr1579, Olr596, Olr583***, *Olr1662, Olr122, Olr1459, Olr255, Olr168;* ***Olr621, Or1423, Olr721, Olr386, Olr27, Olr1673, Olr513, Olr830, Olr1496, Olr1256, Olr127, Olr1539, Olr1249, Olr56, Olr1292, Olr713, Olr237, Olr771, Olr995,*** *Olr287,Olr1201, Olr310, Olr98*)	C-C chemokine binding (***Ccr6, Ccr5,*** *Ackr1*)	Hyperphosphatasia with mental retardation syndrome; Mabry syndrome (***Pigw***, *Pigo*)
	positive regulation of Ras protein signal transduction (***Kras***, ***RGD1560455,***	anaphase-promoting complex binding (***Clspn, Cdc20***)	Autosomal recessive spinocerebellar ataxias

	Gpr55, LOC100362819, Gpr4)		(***Uba5, Coq8b, Ano10, Sptbn2***)
	G-protein coupled receptor signaling pathway (***Adgrv1, Ccr6, Olr1621, LOC679803, Olr1692, Ccr5, Olr1472, Gng10, RGD1560455, Olr551, Fzd5, Gpr55, Olr894, Olr1579, Olr596, Olr583, Fcnb***, *Olr1662, Olr122, Ins2, Olr1459, Ccr1l1, Olr255, Olr168; Vom2r16;* ***Olr621, Vom1r44, Olr1423, Sstr4, Olr721, Gpr4, Camk2b,*** *Olr287,* ***Vom2r26, Olr386, Olr27, Olr1673, Olr513, Gng13, Olr830, Olr1496, Olr1256, Olr127, Olr1539, Olr1249, Olr56, Olr1292, Vom1r42, Mrgprf, Olr713, Olr237, Vom1r32, Olr771, Olr995***, *Olr1201, Olr310, Vom1r53, Olr98*)	olfactory receptor activity (***Olr1621, LOC679803, Olr1692, Olr1472, Olr551, Olr894, Olr1579, Olr596, Olr583***, *Olr1662, Olr122, Olr1459, Olr255, Olr168,* ***Olr621, Olr1423, Olr721, Olr287, Olr386, Olr27, Olr1673, Olr513, Olr830, Olr1496, Olr1256, Olr127, Olr1539, Olr1249, Olr56, Olr1292, Olr713, Olr237, Olr771, Olr995***, *Olr1201, Olr310, Olr98*)	Inherited glycosylphosphatidylinositol deficiencies (***Pigw***, *Pigo*)
	positive regulation of translational elongation (*Eif5a2;* ***Rpl30*)**	C-C chemokine receptor activity (***Ccr6, Ccr5,*** *Ccr1l1*)	Keutel syndrome (***Mgp***)

	Spemann organizer formation (***Fzd5***; ***Wnt3***)	G-protein coupled receptor activity (***Adgrv1, Ccr6***, ***Olr1621***, ***LOC679803***, ***Olr1692, Ccr5***, ***RGD1560455***, ***Fzd5, Gpr55, Olr894, Olr1579, Olr583***, *Olr1662, Olr122, Olr1459, Ccr1l1, Olr255, Olr168, Vom2r16;* ***Vom1r44, Olr1423, Sstr4, Olr721, Gpr4***, ***Vom2r26, Olr386, Olr27, Olr1673, Olr513, Olr830, Olr1496, Olr1256, Olr127, Olr1539, Olr56, Olr1292, Vom1r42, Mrgprf, Olr713, Olr237, Vom1r32, Olr771***, *Olr287, Olr1201*)	Opitz-GBBB syndrome (*Mid1*)
Day 13	mitochondrial tRNA processing (***Trmt10c, Trnt1***)	RNA binding (***Ncl, Tut1, Prpf40a, Crnkl1, Esf1, Eif1a, Magoh, Trmt10c, Zc3h15, Gfm1, Rida, Srek1, Htatsf1, Rpl30, Rsl24d1, Gar1, Tfam, Eif4a2, Trnt1, Zcchc9, Snrpd3, Rpl15, Akap17b, Fam133b,*** *Rpgr, Ngrn, Sap18, Rp137a, Pdia3;* ***Pcbp4, Ass1, Snupn***, *LOC501233, Oas1a, Celf3*)	Transient neonatal diabetes mellitus (***Zfp68, Zfp189, Zfp560, LOC102555644, LOC102552284, LOC102555672, LOC102550291, Zfp141, LOC100910577; Zfp184, Zfp94***)

	positive regulation of substrate-dependent cell migration, cell attachment to substrate (***Fn1***, *Fbln1*)	selenocysteine insertion sequence binding (***Ncl, Rpl30***)	Postaxial polydactyly (***Zfp68, Zfp189, Zfp560, LOC102555644, LOC102552284, LOC102555672, LOC102550291, Zfp141, LOC100910577, Zfp184, Zfp94***)
	negative regulation of transforming growth factor-beta secretion (***Fn1***, *Fbln1*)	C-C chemokine receptor activity (***Ccr9, Ccr1l1, Ccr2***)	Non-syndromic X-linked mental retardation (***Zfp68, Zfp189, Zfp560, LOC102555644, LOC102552284, LOC102555672, LOC102550291, Zfp141, LOC100910577, Zfp184, Zfp94***)
	regulation of alternative mRNA splicing, via spliceosome (***Magoh, Srek1***, *Sap18; Celf3*)	hydrogen-exporting ATPase activity (***Atp6v0e1, Atp6v1g3***, *Atp6ap1l*)	SEMD, Omani type; Spondyloepiphyseal dysplasia with congenital joint dislocations (*Chst3*)
	regulation of vascular endothelial growth factor production (***Noda1, Sulf2, Ccr2***)	cysteine-type endopeptidase inhibitor activity (***LOC689081, Andpro***, *Pttg1, Ngp*)	Hypotrichosis and recurrent skin vesicles (***Dsc3***)
Day 17	negative regulation of oxidoreductase activity (***Cav1, Hp***, *Ins2, Prkn, Tmlhe*)	plus-end directed microfilament motor activity (***Myo10, Myo19***)	Leprosy; Hansen disease (***RT1-T24-1, RT1-M10-1***, *RT1-CE5, RT1-T24-3, RT1-CE15;* ***RT1-CE11***, *Prkn, RT1-CE16*)

	response to mycotoxin (***Lcn2***, *Ass1; **Havcr1***)	scavenger receptor activity (***Tmprss3***, *Loxl3; **Vtn**, Cxcl16*)	Seronegative arthritis (***RT1-T24-1, RT1-M10-1,*** *RT1-CE5, RT1-T24-3, RT1-CE15;* ***RT1-CE11***, *RT1-CE16*)
	negative regulation of fatty acid metabolic process (***Insig2***, *Ins2; Ceacam1*)	oxidoreductase activity, acting on single donors with incorporation of molecular oxygen, incorporation of two atoms of oxygen (***Adi1***, *Tmlhe, Alox12e*)	Ankylosing spondylitis; Bechterew's disease (***RT1-CE11***, *RT1-CE16;* ***RT1-T24-1, RT1-M10-1,*** *RT1-CE5, RT1-T24-3, RT1-CE15*)
	negative regulation by host of viral genome replication (*Prkn; Ceacam1*)	G-protein coupled adenosine receptor activity (***Adora3, Adora2a***)	Stevens-Johnson syndrome and toxic epidermal necrolysis; Lyell's syndrome (***RT1-T24-1, RT1-M10-1***, *RT1-CE5, RT1-T24-3, RT1-CE15;* ***RT1-CE11***, *RT1-CE16*)
	cellular response to amine stimulus (*Ass1; **Htr2b***)	serine-type endopeptidase activity (***Tmprss3, Hp, Prss35***, *Mmp9;* ***Cma1, Try10***, *Prss46*)	Diffuse panbronchiolitis (***RT1-T24-1, RT1-M10-1***, *RT1-CE5, RT1-T24-3, RT1-CE15;* ***RT1-CE11***, *RT1-CE16*)
Day 21	negative regulatory by host of viral genome replication (***Prkn***, *Ceacam1*)	N-acetyl-L-aspartate-L-glutamate ligase activity (***Rimkla***, *LOC100362344*)	Fronto-Otopalatodigital Osteodysplasia (***Flnb***)
	positive regulation of CD8-positive, alpha-beta T cell activation (*Ceacam1;* ***Cd244***)	MHC class I protein binding (***RGD1566006, Cd244, LOC100910669***)	FLNB-related disorders (***Flnb***)

	insulin metabolic process (*Cpe, Ceacam1*)	proton-transporting ATPase activity, rotational mechanism (*Atp6v1f, Atp6ap1l*)	Spondylocarpotarsal synostosis syndrome (***Flnb***)
	negative regulation of leukocyte degranulation (*Ceacam1;* ***Hmox1***)	cytosine deaminase activity (***Apobec1***)	Calpainopathy; Limb-girdle muscular dystrophy 2A (***Capn3***)
	detection of bacterium (*Naip61;* ***Pglyrp4***)	granulocyte colony-stimulating factor receptor binding (*Ceacam1*)	Bart-Pumphrey syndrome (***Gjb2***)

Table S4

Top significant (*p*<0.05) predictive metabolites and lipids in PEPs-exposed rat serum across the different days. For HILIC, samples were normalized using N-benzyl-d5-glycine (IS), 1.10_185.0972 m/z (positive mode) and 1.16_183.0816 m/z (negative mode). For reversed phase LC-MS, samples were normalised to PC (11:0/11:0), 3.22_594.4165 m/z; PG (15:0/15:0), 4.80_717.4709 m/z; Cer (d18:1/17:0), 6.89_534.5274 m/z; DG (19:0/0:0/19:0), 9.12_675.5926 m/z; PC (23:0/23:0), 9.70_930.7865 m/z (positive mode) and to C (17:0), 4.12_269.2484 m/z and PG (15:0/15:0), 4.81_693.4714 m/z (negative mode). Compound identification is based on Human Metabolome Database (HMDB) and Lipid Maps Database. Distribution analysis carried out using PCA and OPLS-DA plots and compound with significant p-value (<0.05) by ANOVA were illustrated.

Day 1 post-exposure

Method	Compounds	ANOVA p-value	Accepted Compound ID	Accepted Description	Adducts	Formula	Score	Mass Error (ppm)	Isotope Similarity
MET-NEG	0.89_141.0909m/z	0.000595	HMDB00392	2-Octenoic acid	M-H	C8H14O2	36.6	-8.50	92.58
MET-NEG	0.93_189.0755m/z	0.000998	HMDB00325	3-Hydroxysuberic acid	M-H	C8H14O5	37.0	-7.25	93.00
MET-POS	0.94_235.1079m/z	0.001494	HMDB02339	5-Methoxytryptophan	M+H	C12H14N2O3	37.7	0.89	89.66
LIPIDS-POS	1.94_357.2804m/z	0.001788	HMDB02007	Tetracosahexaenoic acid	M+H	C24H36O2	38.5	4.39	97.83
LIPIDS-NEG	0.50_632.2037m/z	0.002064	HMDB00825	3'-Sialyllactose	M-H	C23H39NO19	36.1	-0.96	81.68
MET-NEG	0.85_217.1070m/z	0.002320	HMDB00350	3-Hydroxysebacic acid	M-H	C10H18O5	37.2	-5.33	92.15
LIPIDS-POS	2.66_572.3731m/z	0.004015	HMDB10401	LysoPC(22:4(7Z,10Z,13Z,16Z))	M+H	C30H54NO7P	38.9	3.48	98.69
LIPIDS-POS	1.17_512.3380m/z	0.004267	74382709	Scyphostatin A	M+H	C31H45NO5	38.4	1.79	94.33
MET-NEG	0.76_391.2841m/z	0.004374	HMDB00626	Deoxycholic acid	M-H	C24H40O4	39.0	-3.37	98.72
MET-POS	1.02_313.2149m/z	0.005215	HMDB00995	16-Dehydroprogesterone	M+H	C21H28O2	37.7	-4.04	93.20
LIPIDS-POS	1.06_567.3050m/z	0.005298	HMDB30461	Hordatine A	M+H	C28H38N8O5	39.4	2.09	99.28
LIPIDS-POS	1.38_355.2651m/z	0.005392	7851210	5beta-Chola-3,8(14),11-trien-24-oic Acid	M+H	C24H34O2	36.6	5.53	89.31

MET-NEG	4.94_195.0498m/z	0.005627	HMDB00565	Galactonic acid	M-H	C6H12O7	37.2	-6.24	93.05
LIPIDS-POS	1.38_373.2749m/z	0.005887	HMDB13627	Cervonoyl ethanolamide	M+H	C24H36O3	39.1	3.10	99.12
LIPIDS-POS	1.27_567.3053m/z	0.006946	HMDB30461	Hordatine A	M+H	C28H38N8O5	38.4	2.66	94.99
MET-NEG	0.99_243.0614m/z	0.008357	HMDB00296	Uridine	M-H	C9H12N2O6	38.7	-3.47	97.55
LIPIDS-NEG	1.38_407.2794m/z	0.009092	4266387	Cholic acid	M-H	C24H40O5	38.3	-2.15	93.88
MET-POS	1.02_319.2420m/z	0.009920	HMDB31461	Xylene	M+H	C24H30	38.9	0.04	94.69
MET-POS	1.02_247.1692m/z	0.010458	HMDB36207	alpha-Amylcinnamyl acetate	M+H	C16H22O2	39.3	-0.04	96.42
MET-NEG	1.02_407.2795m/z	0.010526	HMDB00619	Cholic acid	M-H	C24H40O5	39.3	-1.87	98.83
MET-POS	3.99_442.3517m/z	0.010531	HMDB13339	3-Hydroxy-11Z-octadecenoylcarnitine	M+H	C25H47NO5	36.6	-2.30	85.52
LIPIDS-POS	1.38_447.2510m/z	0.010971	7850614	PA(19:3(10Z,13Z,16Z)/0:0)	M+H	C22H39O7P	38.0	0.90	91.32
MET-NEG	4.60_196.0240m/z	0.011136	HMDB06955	3-Hydroxy-2-methylpyridine-4,5-dicarboxylate	M-H	C8H7NO5	37.9	-5.64	96.18
MET-NEG	6.43_195.0499m/z	0.011575	HMDB00565	Galactonic acid	M-H	C6H12O7	37.6	-5.78	94.40
MET-NEG	5.25_333.0582m/z	0.011959	HMDB11649	1-(sn-Glycero-3-phospho)-1D-myo-inositol	M-H	C9H19O11P	37.5	-3.10	90.91
MET-NEG	5.24_195.0497m/z	0.012074	HMDB00565	Galactonic acid	M-H	C6H12O7	36.4	-6.98	89.79
MET-POS	0.75_357.2787m/z	0.012395	HMDB02007	Tetracosahexaenoic acid	M+H	C24H36O2	39.6	-0.24	98.44
LIPIDS-POS	8.55_383.3305m/z	0.012482	49703746	25-Dehydrovitamin D3	M+H	C27H42O	37.4	-0.91	88.30
LIPIDS-POS	5.36_854.5723m/z	0.013166	HMDB08452	PC(20:4(5Z,8Z,11Z,14Z)/22:6(4Z,7Z,10Z,13Z,16Z,19Z))	M+H	C50H80NO8P	38.6	3.37	97.18
MET-NEG	3.80_878.5924m/z	0.014434	HMDB61473	PE(DiMe(11,3)/DiMe(13,5))	M-H	C49H86NO10P	37.8	0.82	90.17
MET-NEG	0.77_407.2789m/z	0.014795	HMDB00619	Cholic acid	M-H	C24H40O5	37.9	-3.35	93.34
MET-NEG	0.93_167.0197m/z	0.017266	HMDB00289	Uric acid	M-H	C5H4N4O3	37.5	-8.10	96.65
MET-NEG	6.40_154.0610m/z	0.018180	HMDB00177	L-Histidine	M-H	C6H9N3O2	37.3	-7.86	95.47
MET-NEG	0.87_247.1176m/z	0.021154	HMDB02053	Histidylproline diketopiperazine	M-H	C12H16N4O2	35.8	-9.81	89.98
MET-NEG	0.76_331.1769m/z	0.021609	HMDB28962	Lysyl-Tryptophan	M-H	C17H24N4O3	37.5	-1.91	89.88
LIPIDS-NEG	2.29_500.2775m/z	0.022305	74380436	PE(20:4(5Z,8Z,11Z,14Z)/0:0)	M-H	C25H44NO7P	37.6	-1.62	90.01
LIPIDS-POS	1.85_357.2802m/z	0.022835	HMDB02007	Tetracosahexaenoic acid	M+H	C24H36O2	38.7	3.93	98.31
MET-NEG	5.27_191.0184m/z	0.023486	HMDB00094	Citric acid	M-H	C6H8O7	37.1	-6.97	93.60
MET-NEG	2.47_885.5513m/z	0.023634	HMDB09793	PI(16:0/22:4(10Z,13Z,16Z,19Z))	M-H	C47H83O13P	38.7	1.67	95.58
MET-POS	1.03_273.1833m/z	0.025368	HMDB00151	Estradiol	M+H	C18H24O2	38.4	-5.86	98.58
LIPIDS-POS	5.56_856.5866m/z	0.025923	HMDB08387	PC(20:3(5Z,8Z,11Z)/22:6(4Z,7Z,10Z,13Z,16Z,19Z))	M+H	C50H82NO8P	37.1	1.83	87.80

MET-NEG	0.99_111.0189m/z	0.027265	HMDB00300	Uracil	M-H	C4H4N2O2	37.8	-9.57	99.53
MET-POS	4.42_836.6085m/z	0.029879	123068827	LacCer(d18:0/14:0)	M+H	C44H85NO13	38.9	-1.00	95.70
MET-NEG	4.94_193.0342m/z	0.032333	HMDB02545	Galacturonic acid	M-H	C6H10O7	38.0	-5.95	96.75
MET-NEG	1.57_167.0200m/z	0.035036	HMDB00289	Uric acid	M-H	C5H4N4O3	38.4	-6.54	99.50
MET-POS	1.04_699.5948m/z	0.035258	HMDB07355	DG(18:4(6Z,9Z,12Z,15Z)/24:1(15Z)/0:0)	M+H	C45H78O5	38.4	3.75	96.35
LIPIDS-POS	1.39_704.5207m/z	0.035975	HMDB07870	PC(14:0/16:1(9Z))	M+H	C38H74NO8P	38.1	-2.56	93.39
MET-NEG	0.85_243.1225m/z	0.036818	HMDB32472	Polyethylene, oxidized	M-H	C12H20O5	36.3	-5.18	87.54
MET-NEG	6.19_145.0971m/z	0.037634	HMDB00182	L-Lysine	M-H	C6H14N2O2	37.9	-7.97	98.22
MET-NEG	2.50_861.5500m/z	0.038384	HMDB09786	PI(16:0/20:2(11Z,14Z))	M-H	C45H83O13P	37.8	0.13	89.36
MET-POS	0.66_585.2701m/z	0.040417	HMDB00054	Bilirubin	M+H	C33H36N4O6	38.7	-1.17	94.86
MET-NEG	0.86_261.1332m/z	0.043351	HMDB31897	Phaseolic acid	M-H	C12H22O6	37.7	-4.29	93.53
LIPIDS-POS	6.51_589.5196m/z	0.045289	LMFA07010691	Mayolene-20	M+H	C38H68O4	37.3	0.98	87.84
MET-NEG	4.94_241.0908m/z	0.045687	HMDB29953	D-erythro-D-galacto-octitol	M-H	C8H18O8	38.0	-8.66	99.54
LIPIDS-POS	1.44_660.4945m/z	0.045947	123062599	PE(P-16:0/15:1(9Z))	M+H	C36H70NO7P	38.9	-2.71	97.82
MET-NEG	0.75_405.2638m/z	0.046924	HMDB00502	3-Oxocholic acid	M-H	C24H38O5	38.2	-2.13	93.44
LIPIDS-NEG	2.55_391.2246m/z	0.049008	123060599	CPA(16:0)	M-H	C19H37O6P	36.8	-2.34	86.76
MET-NEG	2.51_833.5195m/z	0.049831	HMDB09784	PI(16:0/18:2(9Z,12Z))	M-H	C43H79O13P	38.8	1.08	95.18
MET-NEG	0.67_421.2243m/z	0.049847	HMDB32386	2-Methylacetophenone	M-H	C20H38O7S	37.7	-5.40	94.59

Day 5 post-exposure

Method	Compounds	ANOVA p-value	Accepted Compound ID	Accepted Description	Adducts	Formula	Score	Mass Error (ppm)	Isotope Similarity
MET-NEG	0.66_433.3312m/z	0.000347	HMDB00359	3a,7a-Dihydroxycoprostanic acid	M-H	C27H46O4	36.7	-2.55	86.43
MET-POS	3.72_766.5728m/z	0.000456	HMDB08194	PC(18:3(6Z,9Z,12Z)/P-18:1(11Z))	M+H	C44H80NO7P	37.4	-2.21	89.45
LIPIDS-POS	6.25_544.3407m/z	0.002871	HMDB10395	LysoPC(20:4(5Z,8Z,11Z,14Z))	M+H	C28H50NO7P	39.1	1.80	97.61
LIPIDS-POS	6.12_568.3407m/z	0.003274	HMDB10404	LysoPC(22:6(4Z,7Z,10Z,13Z,16Z,19Z))	M+H	C30H50NO7P	37.1	1.69	87.55
LIPIDS-POS	6.49_886.6321m/z	0.003338	HMDB08616	PC(22:2(13Z,16Z)/22:6(4Z,7Z,10Z,13Z,16Z,19Z))	M+H	C52H88NO8P	37.6	0.03	87.89
LIPIDS-NEG	0.58_164.0711m/z	0.003991	HMDB00159	L-Phenylalanine	M-H	C9H11NO2	37.8	-3.91	93.57

MET-POS	1.27_267.1338m/z	0.005261	HMDB29005	Phenylalanyl-Threonine	M+H	C13H18N2O4	38.8	-0.52	94.65
MET-NEG	0.66_277.2164m/z	0.005911	HMDB01388	Alpha-Linolenic acid	M-H	C18H30O2	38.0	-3.22	93.90
MET-NEG	0.84_206.0448m/z	0.006551	HMDB00978	4-(2-Aminophenyl)-2,4-dioxobutanoic acid	M-H	C10H9NO4	38.3	-5.31	97.58
LIPIDS-POS	6.31_570.3565m/z	0.008668	HMDB10402	LysoPC(22:5(4Z,7Z,10Z,13Z,16Z))	M+H	C30H52NO7P	39.0	1.95	97.26
LIPIDS-POS	6.69_718.5760m/z	0.008920	HMDB07896	PC(14:0/P-18:0)	M+H	C40H80NO7P	37.7	2.06	91.15
MET-NEG	0.69_297.2422m/z	0.009635	HMDB10736	3-Oxooctadecanoic acid	M-H	C18H34O3	37.0	-4.52	90.23
LIPIDS-NEG	2.76_532.3402m/z	0.009921	HMDB11492	LysoPE(0:0/22:2(13Z,16Z))	M-H	C27H52NO7P	35.7	-1.31	79.95
LIPIDS-POS	5.50_643.5297m/z	0.011455	HMDB07119	DG(16:0/22:5(4Z,7Z,10Z,13Z,16Z)/0:0)	M+H	C41H70O5	38.0	0.15	90.34
LIPIDS-POS	2.76_548.3740m/z	0.012275	HMDB10392	LysoPC(20:2(11Z,14Z))	M+H	C28H54NO7P	38.6	5.34	99.37
MET-POS	4.69_520.3408m/z	0.013100	HMDB10386	LysoPC(18:2(9Z,12Z))	M+H	C26H50NO7P	35.4	2.02	79.29
MET-POS	4.62_548.3698m/z	0.013187	HMDB10392	LysoPC(20:2(11Z,14Z))	M+H	C28H54NO7P	37.9	-2.29	92.34
LIPIDS-NEG	2.24_578.3449m/z	0.014141	123063850	PS(22:1(11Z)/0:0)	M-H	C28H54NO9P	34.3	-2.50	74.33
LIPIDS-POS	3.51_456.4065m/z	0.017643	HMDB06460	Arachidyl carnitine	M+H	C27H53NO4	36.4	3.77	86.26
MET-NEG	4.81_279.2321m/z	0.018014	HMDB00673	Linoleic acid	M-H	C18H32O2	37.9	-2.91	92.97
LIPIDS-POS	7.88_337.2747m/z	0.018687	HMDB06070	Pregnanetriol	M+H	C21H36O3	38.7	2.77	97.01
LIPIDS-POS	10.30_305.2481m/z	0.020290	HMDB01043	Arachidonic acid	M+H	C20H32O2	38.7	1.94	95.78
MET-POS	0.81_135.0806m/z	0.020338	HMDB29697	Cinnamyl alcohol	M+H	C9H10O	38.0	1.52	91.83
MET-POS	4.71_634.2208m/z	0.021047	HMDB00825	3'-Sialyllactose	M+H	C23H39NO19	38.1	3.06	94.16
LIPIDS-POS	2.76_530.3623m/z	0.021158	49703571	2-(8-[3]-ladderane-octanyl)-sn-glycero-3-phosphocholine	M+H	C28H52NO6P	38.2	3.49	95.03
MET-POS	4.87_204.1244m/z	0.022434	HMDB00201	L-Acetylcarnitine	M+H	C9H17NO4	38.4	6.60	99.44
MET-NEG	0.65_329.2460m/z	0.023084	HMDB06528	Docosapentaenoic acid	M-H	C22H34O2	37.2	-7.76	94.75
LIPIDS-POS	5.21_780.5570m/z	0.024399	HMDB07890	PC(14:0/22:5(4Z,7Z,10Z,13Z,16Z))	M+H	C44H78NO8P	36.9	4.08	89.47
LIPIDS-POS	7.90_669.5451m/z	0.024533	HMDB07179	DG(18:0/22:6(4Z,7Z,10Z,13Z,16Z,19Z)/0:0)	M+H	C43H72O5	39.0	-0.21	95.21
LIPIDS-POS	2.75_564.3682m/z	0.024953	7983365	PC(18:1(9E)/2:0)	M+H	C28H54NO8P	38.6	3.98	97.77
LIPIDS-POS	6.31_520.3403m/z	0.025188	HMDB10386	LysoPC(18:2(9Z,12Z))	M+H	C26H50NO7P	39.6	1.01	99.07
LIPIDS-POS	2.28_380.2565m/z	0.025311	HMDB00277	Sphingosine 1-phosphate	M+H	C18H38NO5P	39.1	1.11	97.02
MET-NEG	0.67_445.1884m/z	0.026281	HMDB04483	Estrone glucuronide	M-H	C24H30O8	35.8	3.63	83.08
MET-NEG	1.26_284.0878m/z	0.027198	HMDB05923	N4-Acetylcytidine	M-H	C11H15N3O6	38.2	-3.60	95.25
MET-NEG	0.65_331.2624m/z	0.028226	HMDB02226	Adrenic acid	M-H	C22H36O2	37.3	-5.49	92.62
MET-NEG	4.60_184.0967m/z	0.029598	HMDB06548	Ecgonine	M-H	C9H15NO3	37.3	-6.49	93.71
MET-NEG	0.65_327.2324m/z	0.030269	HMDB02183	Docosahexaenoic acid	M-H	C22H32O2	38.9	-1.71	96.59

MET-NEG	0.66_279.2323m/z	0.030835	HMDB00673	Linoleic acid	M-H	C18H32O2	39.0	-2.38	97.92
MET-NEG	6.40_154.0610m/z	0.030913	HMDB00177	L-Histidine	M-H	C6H9N3O2	37.3	-7.86	95.47
LIPIDS-POS	2.28_532.3397m/z	0.031548	7850608	PC(19:3(10Z,13Z,16Z)/0:0)	M+H	C27H50NO7P	39.0	-0.07	94.95
LIPIDS-POS	10.30_673.5920m/z	0.032411	HMDB06726	CE(20:4(5Z,8Z,11Z,14Z)	M+H	C47H76O2	38.9	0.27	95.01
LIPIDS-POS	10.45_147.1171m/z	0.033357	HMDB61808	(3-Methyl-2-butenyl)-benzene	M+H	C11H14	38.4	2.16	94.37
LIPIDS-POS	2.10_506.3254m/z	0.033423	HMDB11483	LysoPE(0:0/20:2(11Z,14Z))	M+H	C25H48NO7P	38.4	2.56	94.89
MET-NEG	4.81_504.3091m/z	0.033658	HMDB11483	LysoPE(0:0/20:2(11Z,14Z))	M-H	C25H48NO7P	35.4	-1.01	78.31
LIPIDS-POS	2.64_445.2738m/z	0.034229	85292012	sn-3-O-(geranylgeranyl)glycerol 1-phosphate	M+H	C23H41O6P	38.4	5.42	98.21
MET-POS	1.05_123.0553m/z	0.034302	HMDB01406	Niacinamide	M+H	C6H6N2O	39.7	-0.10	98.79
LIPIDS-POS	7.61_589.5209m/z	0.034367	LMFA07010691	Mayolene-20	M+H	C38H68O4	37.3	3.24	90.28
LIPIDS-POS	6.25_766.5772m/z	0.035247	HMDB08194	PC(18:3(6Z,9Z,12Z)/P-18:1(11Z))	M+H	C44H80NO7P	37.1	3.49	89.79
LIPIDS-POS	6.71_771.4135m/z	0.035582	123069263	Spongipregnoloside C	M+H	C39H62O15	36.3	-3.38	85.39
MET-NEG	0.65_281.2471m/z	0.035595	HMDB00207	Oleic acid	M-H	C18H34O2	38.0	-5.46	96.25
LIPIDS-NEG	2.33_578.3455m/z	0.035661	123063850	PS(22:1(11Z)/0:0)	M-H	C28H54NO9P	38.2	-1.48	92.78
LIPIDS-POS	6.31_792.5921m/z	0.035716	HMDB08457	PC(20:4(5Z,8Z,11Z,14Z)/P-18:1(11Z))	M+H	C46H82NO7P	37.2	2.45	89.04
LIPIDS-POS	9.27_727.6278m/z	0.036691	HMDB07529	DG(20:4(5Z,8Z,11Z,14Z)/24:1(15Z)/0:0)	M+H	C47H82O5	37.7	5.93	95.37
LIPIDS-POS	7.01_561.4902m/z	0.037989	123060444	Mayolene-18	M+H	C36H64O4	37.3	4.35	91.68
MET-NEG	4.74_532.3392m/z	0.038442	HMDB11492	LysoPE(0:0/22:2(13Z,16Z))	M-H	C27H52NO7P	32.6	-3.19	66.70
MET-POS	4.57_480.3446m/z	0.038470	HMDB10407	LysoPC(P-16:0)	M+H	C24H50NO6P	39.1	-0.42	95.85
LIPIDS-POS	2.24_520.3421m/z	0.039430	HMDB10386	LysoPC(18:2(9Z,12Z))	M+H	C26H50NO7P	38.8	4.56	99.21
MET-POS	0.65_281.1022m/z	0.040417	HMDB33884	Gravolenic acid	M+H	C14H16O6	39.2	0.88	97.22
MET-POS	4.87_145.0497m/z	0.041946	HMDB00393	3-Hexenedioic acid	M+H	C6H8O4	39.6	1.07	99.51
LIPIDS-POS	6.07_730.5752m/z	0.046401	HMDB07963	PC(15:0/P-18:1(11Z))	M+H	C41H80NO7P	36.4	0.89	83.28
MET-POS	0.79_156.0812m/z	0.048154	HMDB33123	4-Phenylpyridine	M+H	C11H9N	38.5	2.45	95.41
LIPIDS-POS	10.30_147.1171m/z	0.048313	HMDB61808	(3-Methyl-2-butenyl)-benzene	M+H	C11H14	38.8	2.13	96.48
LIPIDS-POS	6.71_665.5155m/z	0.049661	HMDB07266	DG(18:2(9Z,12Z)/22:6(4Z,7Z,10Z,13Z,16Z,19Z)/0:0)	M+H	C43H68O5	39.1	2.35	98.41

Day 9 post-exposure

**Method**	**Compounds**	**ANOVA p-value**	**Accepted Compound ID**	**Accepted Description**	**Adducts**	**Formula**	**Score**	**Mass Error (ppm)**	**Isotope Similarity**

LIPIDS-POS	8.66_664.6632m/z	0.000400	14714478	Cer(d18:1/25:0)	M+H	C43H85NO3	34.5	4.50	77.80
LIPIDS-NEG	6.10_828.5769m/z	0.000463	123062911	PS(17:0/22:2(13Z,16Z))	M-H	C45H84NO10P	35.1	1.10	76.96
LIPIDS-POS	6.08_522.3559m/z	0.000524	HMDB02815	LysoPC(18:1(9Z))	M+H	C26H52NO7P	39.4	1.01	98.12
LIPIDS-NEG	2.36_409.2350m/z	0.000622	14714459	PA(16:0/0:0)	M-H	C19H39O7P	35.9	-2.59	82.66
LIPIDS-POS	8.93_652.6617m/z	0.002064	7850636	Cer(d18:0/24:0)	M+H	C42H85NO3	39.1	2.21	98.23
LIPIDS-NEG	3.10_534.3559m/z	0.002082	123061583	PC(19:1(9Z)/0:0)	M-H	C27H54NO7P	34.7	-1.14	74.65
LIPIDS-POS	2.71_452.3742m/z	0.002148	LMFA07070011	(11Z,14Z)-eicosadienoylcarnitine	M+H	C27H49NO4	38.4	1.78	94.06
LIPIDS-NEG	6.73_405.2658m/z	0.002951	HMDB00502	3-Oxocholic acid	M-H	C24H38O5	35.3	2.78	79.57
LIPIDS-NEG	6.65_830.5929m/z	0.003750	123062910	PS(17:0/22:1(11Z))	M-H	C45H86NO10P	37.3	1.48	88.05
LIPIDS-NEG	6.10_768.5553m/z	0.003998	HMDB07947	PC(15:0/20:3(5Z,8Z,11Z))	M-H	C43H80NO8P	39.1	0.56	96.08
LIPIDS-POS	7.77_868.6799m/z	0.004154	HMDB08158	PC(18:2(9Z,12Z)/24:1(15Z))	M+H	C50H94NO8P	37.0	1.08	86.35
LIPIDS-NEG	4.10_474.3615m/z	0.004423	LMFA07070022	(7Z,10Z,13Z,16Z)-docosatetraenoylcarnitine	M-H	C29H49NO4	35.4	5.43	83.41
MET-POS	3.56_428.3728m/z	0.004615	HMDB00848	Stearoylcarnitine	M+H	C25H49NO4	38.7	-1.58	95.43
LIPIDS-POS	3.04_454.3903m/z	0.004981	LMFA07070010	(11Z)-eicoseneoylcarnitine	M+H	C27H51NO4	38.4	2.79	95.51
LIPIDS-NEG	2.45_435.2508m/z	0.005097	14714461	PA(18:1(9Z)/0:0)	M-H	C21H41O7P	36.3	-2.04	83.78
LIPIDS-POS	2.60_426.3594m/z	0.005708	HMDB05065	Oleoylcarnitine	M+H	C25H47NO4	38.7	3.76	98.09
MET-NEG	0.65_331.2624m/z	0.005715	HMDB02226	Adrenic acid	M-H	C22H36O2	37.3	-5.49	92.62
MET-NEG	0.65_355.2637m/z	0.005762	HMDB02007	Tetracosahexaenoic acid	M-H	C24H36O2	38.7	-1.43	95.36
LIPIDS-POS	2.96_428.3750m/z	0.005823	HMDB00848	Stearoylcarnitine	M+H	C25H49NO4	38.7	3.75	98.12
LIPIDS-POS	8.51_650.6462m/z	0.006259	7850630	Cer(d18:1/24:0)	M+H	C42H83NO3	38.3	2.49	94.70
MET-POS	3.62_400.3426m/z	0.006938	HMDB00222	L-Palmitoylcarnitine	M+H	C23H45NO4	39.4	1.07	98.52
LIPIDS-POS	8.51_668.6578m/z	0.006994	85297405	Cer(d18:0/h24:0)	M+H	C42H85NO4	38.4	4.06	96.78
MET-POS	3.57_426.3578m/z	0.007002	HMDB05065	Oleoylcarnitine	M+H	C25H47NO4	39.2	0.02	96.10
LIPIDS-NEG	6.65_770.5713m/z	0.007415	HMDB07946	PC(15:0/20:2(11Z,14Z))	M-H	C43H82NO8P	39.2	0.97	97.23
LIPIDS-POS	2.01_422.3291m/z	0.008615	HMDB06318	Gamma-linolenyl carnitine	M+H	C25H43NO4	36.9	6.30	91.51
MET-POS	0.65_453.3408m/z	0.008911	HMDB35888	Tyromycic acid	M+H	C30H44O3	36.0	9.80	90.89
LIPIDS-POS	8.01_648.6304m/z	0.009134	7850627	Cer(d18:1/24:1(15Z))	M+H	C42H81NO3	36.7	2.33	86.41
LIPIDS-POS	2.50_400.3439m/z	0.009230	HMDB00222	L-Palmitoylcarnitine	M+H	C23H45NO4	38.5	4.34	97.38
LIPIDS-POS	7.90_669.5451m/z	0.009953	HMDB07179	DG(18:0/22:6(4Z,7Z,10Z,13Z,16Z,19Z)/0:0)	M+H	C43H72O5	39.0	-0.21	95.21
LIPIDS-NEG	3.51_446.3302m/z	0.010225	74380333	N-docosanoyl taurine	M-H	C24H49NO4S	34.6	-1.78	75.34
LIPIDS-POS	8.47_624.6303m/z	0.011302	7850634	Cer(d18:0/22:0)	M+H	C40H81NO3	38.8	2.19	96.67
LIPIDS-POS	8.78_632.6373m/z	0.011722	173737522	Cer(m18:1(4E)/24:1(15Z))	M+H	C42H81NO2	38.4	5.26	98.11

LIPIDS-POS	8.28_870.6946m/z	0.011786	HMDB08092	PC(18:1(11Z)/24:1(15Z))	M+H	C50H96NO8P	37.4	-0.08	86.89
MET-NEG	5.27_191.0184m/z	0.013623	HMDB00094	Citric acid	M-H	C6H8O7	37.1	-6.97	93.60
LIPIDS-POS	10.73_147.1171m/z	0.013865	HMDB61808	(3-Methyl-2-butenyl)-benzene	M+H	C11H14	38.2	2.19	93.42
LIPIDS-POS	6.84_287.2386m/z	0.014056	HMDB00305	Vitamin A	M+H	C20H30O	37.5	5.76	93.94
LIPIDS-NEG	5.40_909.5527m/z	0.015119	HMDB09821	PI(18:0/22:6(4Z,7Z,10Z,13Z,16Z,19Z))	M-H	C49H83O13P	38.9	3.13	98.27
MET-NEG	4.74_532.3392m/z	0.015330	HMDB11492	LysoPE(0:0/22:2(13Z,16Z))	M-H	C27H52NO7P	32.6	-3.19	66.70
MET-POS	0.66_585.2701m/z	0.015672	HMDB00054	Bilirubin	M+H	C33H36N4O6	38.7	-1.17	94.86
MET-NEG	0.65_327.2324m/z	0.015950	HMDB02183	Docosahexaenoic acid	M-H	C22H32O2	38.9	-1.71	96.59
MET-NEG	5.25_333.0582m/z	0.016279	HMDB11649	1-(sn-Glycero-3-phospho)-1D-myo-inositol	M-H	C9H19O11P	37.5	-3.10	90.91
MET-POS	4.55_578.4178m/z	0.016504	HMDB10399	LysoPC(22:1(13Z))	M+H	C30H60NO7P	38.6	-0.44	93.37
LIPIDS-POS	8.95_678.6759m/z	0.017091	HMDB04955	Cer(d18:1/26:0)	M+H	C44H87NO3	38.3	-0.02	91.54
LIPIDS-NEG	2.76_532.3402m/z	0.017201	HMDB11492	LysoPE(0:0/22:2(13Z,16Z))	M-H	C27H52NO7P	35.7	-1.31	79.95
LIPIDS-NEG	3.23_327.2325m/z	0.017205	HMDB02183	Docosahexaenoic acid	M-H	C22H32O2	38.6	-1.40	94.90
LIPIDS-NEG	0.58_271.0699m/z	0.017206	HMDB61177	Pyridine N-oxide glucuronide	M-H	C11H14NO7+	38.5	0.67	93.46
LIPIDS-NEG	2.33_578.3455m/z	0.017729	123063850	PS(22:1(11Z)/0:0)	M-H	C28H54NO9P	38.2	-1.48	92.78
LIPIDS-POS	8.47_606.6185m/z	0.017974	173737519	Cer(m18:1(4E)/22:0)	M+H	C40H79NO2	37.6	0.18	88.25
LIPIDS-NEG	5.59_790.5399m/z	0.018177	HMDB07958	PC(15:0/22:6(4Z,7Z,10Z,13Z,16Z,19Z))	M-H	C45H78NO8P	37.5	0.80	88.36
LIPIDS-POS	6.23_772.5875m/z	0.018605	HMDB07946	PC(15:0/20:2(11Z,14Z))	M+H	C43H82NO8P	38.5	3.17	96.18
LIPIDS-POS	5.15_742.5399m/z	0.019559	HMDB07941	PC(15:0/18:3(6Z,9Z,12Z))	M+H	C41H76NO8P	37.6	2.32	90.53
LIPIDS-POS	2.75_570.3574m/z	0.019857	HMDB10402	LysoPC(22:5(4Z,7Z,10Z,13Z,16Z))	M+H	C30H52NO7P	37.9	3.50	93.74
LIPIDS-POS	8.51_826.6682m/z	0.020290	HMDB08588	PC(22:1(13Z)/P-18:1(11Z))	M+H	C48H92NO7P	37.7	-0.25	88.65
LIPIDS-POS	7.78_842.6654m/z	0.020552	HMDB08026	PC(16:1(9Z)/24:1(15Z))	M+H	C48H92NO8P	38.8	2.49	97.20
LIPIDS-POS	8.74_664.6620m/z	0.020767	14714478	Cer(d18:1/25:0)	M+H	C43H85NO3	31.6	2.72	60.99
LIPIDS-POS	6.83_800.6197m/z	0.021078	HMDB07954	PC(15:0/22:2(13Z,16Z))	M+H	C45H86NO8P	37.5	4.18	92.15
LIPIDS-NEG	2.75_466.3288m/z	0.021390	14713946	PC(O-15:0/0:0)	M-H	C23H50NO6P	37.0	-3.15	88.69
LIPIDS-POS	1.72_283.2054m/z	0.021489	HMDB35695	Vitamin A2 aldehyde	M+H	C20H26O	37.9	-0.91	90.51
LIPIDS-POS	8.95_696.6877m/z	0.022123	85297406	Cer(d18:0/h26:0)	M+H	C44H89NO4	38.8	1.78	96.07
MET-NEG	0.66_575.4663m/z	0.022174	HMDB07075	DG(15:0/18:3(6Z,9Z,12Z)/0:0)	M-H	C36H64O5	36.5	-3.06	86.24
LIPIDS-NEG	6.21_768.5550m/z	0.022180	HMDB07947	PC(15:0/20:3(5Z,8Z,11Z))	M-H	C43H80NO8P	37.3	0.15	86.67
LIPIDS-POS	10.73_369.3529m/z	0.023494	49703700	3-Deoxyvitamin D3	M+H	C27H44	38.9	3.70	99.07

LIPIDS-POS	6.85_361.2739m/z	0.024234	14715028	1alpha,23-dihydroxy-24,25,26,27-tetranorvitamin D3 / 1alpha,23-dihydroxy-24,25,26,27-tetranorcholecalciferol	M+H	C23H36O3	38.6	0.53	93.67
MET-POS	5.69_518.3215m/z	0.024389	HMDB10387	LysoPC(18:3(6Z,9Z,12Z))	M+H	C26H48NO7P	37.9	-4.99	95.13
MET-POS	3.69_146.0604m/z	0.024773	HMDB29737	1H-Indole-3-carboxaldehyde	M+H	C9H7NO	39.3	2.32	99.26
LIPIDS-POS	8.01_640.6255m/z	0.024884	160780160	Cer(d18:0/22:0(2OH))	M+H	C40H81NO4	38.7	2.67	96.47
LIPIDS-NEG	2.22_597.3040m/z	0.025122	74380468	PI(18:1(9Z)/0:0)	M-H	C27H51O12P	34.6	-0.86	74.29
LIPIDS-POS	3.52_403.3574m/z	0.025481	HMDB01419	24-Hydroxycholesterol	M+H	C27H46O2	38.4	0.86	93.00
LIPIDS-POS	3.09_566.3834m/z	0.025570	7982867	PC(10:0/10:0)	M+H	C28H56NO8P	38.8	3.06	97.54
LIPIDS-NEG	2.17_433.2352m/z	0.025921	123067332	PA(18:2(9Z,12Z)/0:0)	M-H	C21H39O7P	38.0	-2.05	92.25
LIPIDS-NEG	6.07_889.5836m/z	0.026069	HMDB09791	PI(16:0/22:2(13Z,16Z))	M-H	C47H87O13P	38.1	2.70	93.51
MET-POS	3.60_424.3422m/z	0.026464	HMDB06469	Linoleyl carnitine	M+H	C25H45NO4	39.0	0.11	95.33
LIPIDS-POS	7.16_814.6364m/z	0.026609	HMDB07927	PC(14:1(9Z)/24:1(15Z))	M+H	C46H88NO8P	38.2	5.32	97.29
LIPIDS-POS	2.14_416.3389m/z	0.026938	HMDB13336	3-Hydroxyhexadecanoylcarnitine	M+H	C23H45NO5	34.2	4.52	76.49
MET-POS	3.70_118.0651m/z	0.027147	HMDB00738	Indole	M+H	C8H7N	39.7	0.02	98.63
MET-POS	3.86_496.3395m/z	0.027697	HMDB10382	LysoPC(16:0)	M+H	C24H50NO7P	38.8	-0.49	94.38
LIPIDS-POS	5.25_445.3679m/z	0.027742	HMDB11662	3-beta-Hydroxy-4-beta-methyl-5-alpha-cholest-7-ene-4-alpha-carboxylate	M+H	C29H48O3	39.1	0.58	96.40
MET-POS	3.69_188.0714m/z	0.028496	HMDB00734	Indoleacrylic acid	M+H	C11H9NO2	38.8	4.46	99.29
LIPIDS-POS	2.08_407.3162m/z	0.028498	LMST04070032	Homochenodeoxycholic acid	M+H	C25H42O4	37.6	1.48	89.80
MET-POS	1.06_220.1190m/z	0.028879	HMDB00210	Pantothenic acid	M+H	C9H17NO5	36.7	4.84	89.29
MET-POS	3.69_132.0809m/z	0.028908	HMDB00466	3-Methylindole	M+H	C9H9N	38.3	1.05	92.90
MET-POS	3.78_782.5699m/z	0.029404	HMDB07889	PC(14:0/22:4(7Z,10Z,13Z,16Z))	M+H	C44H80NO8P	35.2	0.59	76.83
LIPIDS-NEG	6.02_742.5398m/z	0.029460	HMDB07940	PC(15:0/18:2(9Z,12Z))	M-H	C41H78NO8P	39.6	0.78	98.91
LIPIDS-NEG	6.02_816.5757m/z	0.030889	123062864	PS(16:0/22:1(11Z))	M-H	C44H84NO10P	35.3	-0.40	77.12
MET-POS	1.30_342.1397m/z	0.031769	HMDB06591	Lactosamine	M+H	C12H23NO10	38.3	0.74	92.41
LIPIDS-POS	8.71_638.6462m/z	0.032127	HMDB11767	Cer(d18:0/23:0)	M+H	C41H83NO3	38.3	2.63	94.66
LIPIDS-POS	10.58_632.6341m/z	0.032201	173737522	Cer(m18:1(4E)/24:1(15Z))	M+H	C42H81NO2	39.1	0.16	95.49
MET-POS	3.71_160.0763m/z	0.032441	HMDB01190	Indoleacetaldehyde	M+H	C10H9NO	37.4	3.65	91.40
LIPIDS-POS	2.76_548.3740m/z	0.032471	HMDB10392	LysoPC(20:2(11Z,14Z))	M+H	C28H54NO7P	38.6	5.34	99.37
MET-POS	3.59_414.3574m/z	0.033191	HMDB06210	Heptadecanoyl carnitine	M+H	C24H47NO4	36.7	-0.85	84.38
MET-NEG	1.11_218.1025m/z	0.033636	HMDB00210	Pantothenic acid	M-H	C9H17NO5	38.7	-3.94	98.11

MET-POS	4.55_98.9840m/z	0.034332	HMDB02142	Phosphoric acid	M+H	H3O4P	39.3	-2.21	99.00
MET-POS	3.70_144.0810m/z	0.034393	HMDB33115	6-Methylquinoline	M+H	C10H9N	39.3	1.26	98.11
LIPIDS-NEG	4.64_431.3887m/z	0.035563	123060225	29:3(5Z,9Z,23Z)	M-H	C29H52O2	35.6	-1.81	80.06
LIPIDS-NEG	4.80_881.5206m/z	0.036026	123065030	PI(16:0/22:6(4Z,7Z,10Z,13Z,16Z,19Z))	M-H	C47H79O13P	38.4	2.36	95.01
MET-POS	1.04_699.5948m/z	0.036172	HMDB07355	DG(18:4(6Z,9Z,12Z,15Z)/24:1(15Z)/0:0)	M+H	C45H78O5	38.4	3.75	96.35
LIPIDS-POS	6.17_796.5835m/z	0.036220	HMDB07955	PC(15:0/22:4(7Z,10Z,13Z,16Z))	M+H	C45H82NO8P	37.4	-2.05	89.56
LIPIDS-POS	2.72_510.3579m/z	0.036740	HMDB11481	LysoPE(0:0/20:0)	M+H	C25H52NO7P	38.6	4.90	98.91
MET-NEG	3.87_766.5395m/z	0.037428	HMDB07949	PC(15:0/20:4(5Z,8Z,11Z,14Z))	M-H	C43H78NO8P	34.9	0.30	75.02
MET-NEG	0.66_419.3512m/z	0.037901	HMDB01457	5-b-Cholestane-3a ,7a ,12a-triol	M-H	C27H48O3	34.6	-4.45	78.12
MET-NEG	3.82_203.0815m/z	0.038548	HMDB00929	L-Tryptophan	M-H	C11H12N2O2	38.5	-5.29	98.50
MET-NEG	0.71_413.2182m/z	0.039151	HMDB30895	(4R,5S,7R,11x)-11,12-Dihydroxy-1(10)-spirovetiven-2-one 12-glucoside	M-H	C21H34O8	38.8	0.19	94.00
LIPIDS-POS	3.19_520.3756m/z	0.039265	74380419	PC(P-19:1(12Z)/0:0)	M+H	C27H54NO6P	36.7	-1.15	85.12
LIPIDS-POS	7.22_639.4972m/z	0.039567	HMDB07150	DG(16:1(9Z)/22:6(4Z,7Z,10Z,13Z,16Z,19Z)/0:0)	M+H	C41H66O5	37.9	-1.72	91.40
LIPIDS-NEG	5.97_826.5616m/z	0.040541	123062940	PS(17:1(9Z)/22:2(13Z,16Z))	M-H	C45H82NO10P	37.9	1.50	91.14
MET-POS	4.71_634.2208m/z	0.041084	HMDB00825	3'-Sialyllactose	M+H	C23H39NO19	38.1	3.06	94.16
MET-NEG	1.27_190.0495m/z	0.042248	HMDB00763	5-Hydroxyindoleacetic acid	M-H	C10H9NO3	38.0	-7.88	98.82
LIPIDS-POS	2.56_258.1110m/z	0.042291	HMDB00086	Glycerophosphocholine	M+H	C8H20NO6P	38.4	3.68	96.53
LIPIDS-POS	2.76_530.3623m/z	0.042432	49703571	2-(8-[3]-ladderane-octanyl)-sn-glycero-3-phosphocholine	M+H	C28H52NO6P	38.2	3.49	95.03
LIPIDS-NEG	6.48_804.5770m/z	0.042697	123062821	PS(15:0/22:0)	M-H	C43H84NO10P	33.7	1.17	69.96
LIPIDS-NEG	5.35_835.5356m/z	0.042781	4266294	PI(16:0/18:1(9Z))	M-H	C43H81O13P	38.7	1.72	95.47
LIPIDS-POS	1.72_301.2169m/z	0.043611	HMDB01852	All-trans-retinoic acid	M+H	C20H28O2	39.1	2.46	98.34
LIPIDS-POS	4.95_590.4554m/z	0.043652	160780188	CerP(d18:1/14:0)	M+H	C32H64NO6P	38.8	1.67	95.82
LIPIDS-POS	3.30_520.3760m/z	0.043880	74380419	PC(P-19:1(12Z)/0:0)	M+H	C27H54NO6P	38.0	-0.38	90.70
LIPIDS-NEG	3.03_283.2641m/z	0.044321	HMDB00827	Stearic acid	M-H	C18H36O2	38.3	-0.52	91.97
LIPIDS-POS	2.16_457.2350m/z	0.044716	123067348	PA(20:5(5Z,8Z,11Z,14Z,17Z)/0:0)	M+H	C23H37O7P	39.2	0.12	95.97
LIPIDS-NEG	6.38_718.5390m/z	0.044862	HMDB07935	PC(15:0/16:0)	M-H	C39H78NO8P	35.9	-0.31	79.88
LIPIDS-NEG	6.02_802.5613m/z	0.045028	123062983	PS(18:0/19:1(9Z))	M-H	C43H82NO10P	38.5	1.14	93.94
LIPIDS-NEG	0.85_498.2885m/z	0.045436	HMDB00874	Tauroursodeoxycholic acid	M-H	C26H45NO6S	38.2	-1.93	93.44
MET-NEG	4.82_255.2321m/z	0.045908	HMDB00220	Palmitic acid	M-H	C16H32O2	38.3	-3.36	95.47
MET-NEG	1.23_243.1872m/z	0.046524	HMDB15640	Xylometazoline	M-H	C16H24N2	36.2	2.21	83.71

MET-NEG	1.23_243.0615m/z	0.047008	HMDB00296	Uridine	M-H	C9H12N2O6	38.8	-3.15	97.63
LIPIDS-NEG	2.16_362.2365m/z	0.047024	74380330	N-palmitoyl taurine	M-H	C18H37NO4S	35.6	-1.66	79.99
MET-POS	3.99_442.3517m/z	0.047145	HMDB13339	3-Hydroxy-11Z-octadecenoylcarnitine	M+H	C25H47NO5	36.6	-2.30	85.52
LIPIDS-POS	7.88_337.2747m/z	0.047408	HMDB06070	Pregnanetriol	M+H	C21H36O3	38.7	2.77	97.01
LIPIDS-NEG	3.03_508.3405m/z	0.048065	HMDB11481	LysoPE(0:0/20:0)	M-H	C25H52NO7P	39.7	-0.69	99.15
LIPIDS-NEG	2.56_599.3200m/z	0.048178	74380467	PI(18:0/0:0)	M-H	C27H53O12P	39.6	-0.32	98.18
LIPIDS-POS	7.50_667.5295m/z	0.048375	HMDB07208	DG(18:1(11Z)/22:6(4Z,7Z,10Z,13Z,16Z,19Z)/0:0)	M+H	C43H70O5	37.4	-0.16	87.00
MET-NEG	0.75_229.1435m/z	0.048937	HMDB00623	Dodecanedioic acid	M-H	C12H22O4	37.5	-4.44	92.66
LIPIDS-POS	7.50_645.5467m/z	0.049671	HMDB07118	DG(16:0/22:4(7Z,10Z,13Z,16Z)/0:0)	M+H	C41H72O5	37.7	2.29	91.35
LIPIDS-POS	3.77_645.3994m/z	0.049938	HMDB35978	O-Methylganoderic acid O	M+H	C37H56O9	39.5	-0.41	98.19

Day 13 post-exposure

**Method**	**Compounds**	**ANOVA p-value**	**Accepted Compound ID**	**Accepted Description**	**Adducts**	**Formula**	**Score**	**Mass Error (ppm)**	**Isotope Similarity**
LIPIDS-POS	6.69_812.6190m/z	0.002199	HMDB08020	PC(16:1(9Z)/22:2(13Z,16Z))	M+H	C46H86NO8P	37.4	3.28	90.67
MET-NEG	0.44_203.0008m/z	0.004676	HMDB60013	O-methoxycatechol-O-sulphate	M-H	C7H8O5S	38.2	-5.50	97.41
LIPIDS-POS	5.55_935.5627m/z	0.005977	123065575	PI(20:2(11Z,14Z)/22:6(4Z,7Z,10Z,13Z,16Z,19Z))	M+H	C51H83O13P	38.5	-1.82	94.53
LIPIDS-POS	6.43_706.5757m/z	0.007800	123061419	PC(O-16:0/15:0)	M+H	C39H80NO7P	37.7	1.67	90.59
MET-NEG	4.61_195.0500m/z	0.008734	HMDB00565	Galactonic acid	M-H	C6H12O7	36.3	-5.18	87.41
LIPIDS-NEG	0.74_186.0552m/z	0.009471	HMDB00734	Indoleacrylic acid	M-H	C11H9NO2	35.2	-4.51	81.44
MET-NEG	4.94_195.0498m/z	0.009643	HMDB00565	Galactonic acid	M-H	C6H12O7	37.2	-6.24	93.05
LIPIDS-POS	4.79_687.5450m/z	0.009703	123068747	SM(d18:2/15:0)	M+H	C38H75N2O6P	37.8	2.04	91.68
MET-NEG	1.35_383.0646m/z	0.011704	HMDB40571	2-O-Feruloylhydroxycitric acid	M-H	C16H16O11	37.4	6.72	94.63
LIPIDS-NEG	6.08_746.5133m/z	0.012048	HMDB05780	PE(O-16:1(1Z)/22:6(4Z,7Z,10Z,13Z,16Z,19Z))	M-H	C43H74NO7P	34.9	0.37	75.16
LIPIDS-POS	7.20_864.6489m/z	0.014204	HMDB08257	PC(18:4(6Z,9Z,12Z,15Z)/24:1(15Z))	M+H	C50H90NO8P	38.9	1.46	96.10
MET-POS	0.74_417.3355m/z	0.016009	HMDB06226	24R,25-Dihydroxyvitamin D3	M+H	C27H44O3	37.6	-2.02	90.22
LIPIDS-POS	7.18_838.6349m/z	0.016659	HMDB08054	PC(18:0/22:4(7Z,10Z,13Z,16Z))	M+H	C48H88NO8P	33.2	3.47	70.05
LIPIDS-POS	6.13_740.5239m/z	0.017158	HMDB07943	PC(15:0/18:4(6Z,9Z,12Z,15Z))	M+H	C41H74NO8P	37.6	1.95	90.15
LIPIDS-POS	6.04_830.5680m/z	0.018069	HMDB08156	PC(18:2(9Z,12Z)/22:6(4Z,7Z,10Z,13Z,16Z,19Z))	M+H	C48H80NO8P	36.8	-1.69	85.86

MET-POS	0.69_401.3413m/z	0.018265	HMDB06721	5,6-trans-25-Hydroxyvitamin D3	M+H	C27H44O2	38.8	-0.21	94.44
MET-POS	3.69_132.0809m/z	0.018378	HMDB00466	3-Methylindole	M+H	C9H9N	38.3	1.05	92.90
LIPIDS-POS	5.95_804.5531m/z	0.018972	HMDB08023	PC(16:1(9Z)/22:6(4Z,7Z,10Z,13Z,16Z,19Z))	M+H	C46H78NO8P	37.6	-0.89	89.20
MET-NEG	5.49_173.0918m/z	0.019914	HMDB03357	N-Acetylornithine	M-H	C7H14N2O3	36.5	-7.68	91.31
LIPIDS-NEG	2.30_378.2408m/z	0.021881	4266347	Sphingosine-1-phosphate	M-H	C18H38NO5P	38.6	-1.76	94.97
MET-POS	2.41_282.2798m/z	0.022430	HMDB02117	Oleamide	M+H	C18H35NO	38.7	2.47	96.56
LIPIDS-POS	7.89_776.6544m/z	0.022572	HMDB13406	PC(o-16:0/20:0)	M+H	C44H90NO7P	37.7	2.11	90.89
MET-NEG	0.68_300.0386m/z	0.023478	HMDB00814	N-Acetylglucosamine 6-sulfate	M-H	C8H15NO9S	38.6	-2.80	96.41
MET-NEG	6.43_195.0499m/z	0.023659	HMDB00565	Galactonic acid	M-H	C6H12O7	37.6	-5.78	94.40
LIPIDS-POS	2.10_417.3381m/z	0.023696	HMDB00430	24,25-Dihydroxyvitamin D	M+H	C27H44O3	37.7	4.32	93.45
LIPIDS-NEG	5.64_766.5395m/z	0.025110	HMDB07949	PC(15:0/20:4(5Z,8Z,11Z,14Z))	M-H	C43H78NO8P	38.8	0.30	94.33
LIPIDS-NEG	2.03_325.1839m/z	0.025192	HMDB31031	2-Dodecylbenzenesulfonic acid	M-H	C18H30O3S	35.6	-1.33	79.83
LIPIDS-NEG	2.33_578.3455m/z	0.025882	123063850	PS(22:1(11Z)/0:0)	M-H	C28H54NO9P	38.2	-1.48	92.78
MET-NEG	0.70_313.2371m/z	0.025899	HMDB00782	Octadecanedioic acid	M-H	C18H34O4	37.3	-4.10	91.07
LIPIDS-POS	6.76_768.5562m/z	0.027031	HMDB07949	PC(15:0/20:4(5Z,8Z,11Z,14Z))	M+H	C43H78NO8P	37.5	3.18	91.05
LIPIDS-NEG	0.52_300.0391m/z	0.027154	HMDB00781	N-Acetylgalactosamine 4-sulphate	M-H	C8H15NO9S	38.5	-1.34	94.15
LIPIDS-POS	6.33_822.6042m/z	0.027234	HMDB09212	PE(18:4(6Z,9Z,12Z,15Z)/24:1(15Z))	M+H	C47H84NO8P	36.7	4.17	88.27
LIPIDS-NEG	6.22_764.5240m/z	0.030787	HMDB07951	PC(15:0/20:5(5Z,8Z,11Z,14Z,17Z))	M-H	C43H76NO8P	38.2	0.49	91.57
MET-POS	0.74_277.1034m/z	0.031329	HMDB11737	Gamma Glutamylglutamic acid	M+H	C10H16N2O7	39.1	1.35	97.24
LIPIDS-POS	6.17_796.5835m/z	0.032157	HMDB07955	PC(15:0/22:4(7Z,10Z,13Z,16Z))	M+H	C45H82NO8P	37.4	-2.05	89.56
LIPIDS-NEG	6.46_722.5130m/z	0.033504	HMDB09149	PE(18:3(6Z,9Z,12Z)/P-18:1(11Z))	M-H	C41H74NO7P	38.1	0.01	90.71
LIPIDS-NEG	6.14_738.5083m/z	0.035123	HMDB07943	PC(15:0/18:4(6Z,9Z,12Z,15Z))	M-H	C41H74NO8P	38.9	0.46	95.00
LIPIDS-POS	6.03_717.5918m/z	0.035387	123068755	SM(d18:1/17:0)	M+H	C40H81N2O6P	38.5	1.86	94.68
LIPIDS-POS	6.69_862.6325m/z	0.035830	HMDB08288	PC(20:0/22:6(4Z,7Z,10Z,13Z,16Z,19Z))	M+H	C50H88NO8P	38.5	0.59	93.26
MET-NEG	1.47_512.2676m/z	0.036000	HMDB02639	Sulfolithocholylglycine	M-H	C26H43NO7S	37.9	-2.23	92.10
MET-POS	3.78_782.5699m/z	0.036833	HMDB07889	PC(14:0/22:4(7Z,10Z,13Z,16Z))	M+H	C44H80NO8P	35.2	0.59	76.83
LIPIDS-POS	6.80_766.5382m/z	0.037930	HMDB07951	PC(15:0/20:5(5Z,8Z,11Z,14Z,17Z))	M+H	C43H76NO8P	37.7	0.15	88.56
LIPIDS-POS	2.03_431.3167m/z	0.037985	HMDB11560	MG(0:0/24:6(6Z,9Z,12Z,15Z,18Z,21Z)/0:0)	M+H	C27H42O4	39.1	2.64	98.51
LIPIDS-POS	2.38_380.2567m/z	0.038579	HMDB00277	Sphingosine 1-phosphate	M+H	C18H38NO5P	38.8	1.65	96.14
MET-POS	1.15_429.3712m/z	0.039843	HMDB03822	Cholesteryl acetate	M+H	C29H48O2	37.6	-3.55	92.42
LIPIDS-NEG	6.52_748.5287m/z	0.039958	HMDB09413	PE(20:4(5Z,8Z,11Z,14Z)/P-18:1(11Z))	M-H	C43H76NO7P	38.8	0.07	93.88
MET-NEG	2.26_256.0928m/z	0.040036	HMDB00982	5-Methylcytidine	M-H	C10H15N3O5	37.4	-4.13	91.94

LIPIDS-POS	6.52_361.2742m/z	0.042828	14715028	1alpha,23-dihydroxy-24,25,26,27-tetranorvitamin D3 / 1alpha,23-dihydroxy-24,25,26,27-tetranorcholecalciferol	M+H	C23H36O3	38.6	1.40	94.89
LIPIDS-NEG	2.45_378.2407m/z	0.042915	4266347	Sphingosine-1-phosphate	M-H	C18H38NO5P	36.1	-2.06	82.94
MET-NEG	1.25_551.3586m/z	0.044721	HMDB36298	Cyclopassifloic acid E	M-H	C31H52O8	39.5	-0.69	98.33
LIPIDS-POS	6.44_724.5271m/z	0.045484	HMDB09149	PE(18:3(6Z,9Z,12Z)/P-18:1(11Z))	M+H	C41H74NO7P	39.0	-0.58	95.80
LIPIDS-NEG	5.58_810.5297m/z	0.046888	HMDB10165	PS(18:0/20:4(8Z,11Z,14Z,17Z))	M-H	C44H78NO10P	35.4	0.84	78.07
MET-POS	4.71_634.2208m/z	0.047408	HMDB00825	3'-Sialyllactose	M+H	C23H39NO19	38.1	3.06	94.16
LIPIDS-POS	6.00_758.5736m/z	0.049275	HMDB07880	PC(14:0/20:2(11Z,14Z))	M+H	C42H80NO8P	38.6	5.45	99.03

Day 17 post-exposure

**Method**	**Compounds**	**ANOVA p-value**	**Accepted Compound ID**	**Accepted Description**	**Adducts**	**Formula**	**Score**	**Mass Error (ppm)**	**Isotope Similarity**
LIPIDS-POS	4.96_634.4818m/z	0.000234	HMDB29220	lysoPC(26:1(5Z))	M+H	C34H68NO7P	38.6	1.91	95.31
LIPIDS-NEG	6.10_913.5836m/z	0.000407	HMDB09817	PI(18:0/22:4(10Z,13Z,16Z,19Z))	M-H	C49H87O13P	35.8	2.63	82.27
LIPIDS-NEG	2.33_415.2245m/z	0.001145	HMDB07007	CPA(18:2(9Z,12Z)/0:0)	M-H	C21H37O6P	35.9	-2.28	82.04
MET-NEG	0.76_189.0756m/z	0.001196	HMDB00325	3-Hydroxysuberic acid	M-H	C8H14O5	36.9	-6.50	91.93
LIPIDS-POS	5.21_780.5570m/z	0.001201	HMDB07890	PC(14:0/22:5(4Z,7Z,10Z,13Z,16Z))	M+H	C44H78NO8P	36.9	4.08	89.47
MET-NEG	4.81_504.3091m/z	0.001430	HMDB11483	LysoPE(0:0/20:2(11Z,14Z))	M-H	C25H48NO7P	35.4	-1.01	78.31
LIPIDS-NEG	5.02_859.5359m/z	0.001539	123065123	PI(14:1(9Z)/22:2(13Z,16Z))	M-H	C45H81O13P	39.3	2.01	98.99
LIPIDS-NEG	2.33_505.2548m/z	0.001646	14715089	(22E)-26,26,26,27,27,27-hexafluoro-25-hydroxy-22,23-didehydrovitamin D3 / (22E)-26,26,26,27,27,27-hexafluoro-25-hydroxy-22,23-didehydrocholecalciferol	M-H	C27H36F6O2	38.4	0.19	92.28
LIPIDS-POS	2.24_520.3421m/z	0.001674	HMDB10386	LysoPC(18:2(9Z,12Z))	M+H	C26H50NO7P	38.8	4.56	99.21
MET-NEG	4.81_279.2321m/z	0.001846	HMDB00673	Linoleic acid	M-H	C18H32O2	37.9	-2.91	92.97
LIPIDS-NEG	5.64_766.5395m/z	0.001911	HMDB07949	PC(15:0/20:4(5Z,8Z,11Z,14Z))	M-H	C43H78NO8P	38.8	0.30	94.33
LIPIDS-POS	4.80_634.4794m/z	0.001948	HMDB29220	lysoPC(26:1(5Z))	M+H	C34H68NO7P	37.8	-1.85	91.41
LIPIDS-NEG	4.60_857.5198m/z	0.002024	HMDB09789	PI(16:0/20:4(5Z,8Z,11Z,14Z))	M-H	C45H79O13P	37.7	1.49	90.36
LIPIDS-NEG	2.33_578.3455m/z	0.002270	123063850	PS(22:1(11Z)/0:0)	M-H	C28H54NO9P	38.2	-1.48	92.78

LIPIDS-NEG	2.33_504.3093m/z	0.002484	123062694	PE(20:2(11Z,14Z)/0:0)	M-H	C25H48NO7P	39.7	-0.61	99.19
LIPIDS-POS	5.63_782.5724m/z	0.003433	HMDB07889	PC(14:0/22:4(7Z,10Z,13Z,16Z))	M+H	C44H80NO8P	38.8	3.81	98.26
LIPIDS-NEG	6.10_828.5769m/z	0.003533	123062911	PS(17:0/22:2(13Z,16Z))	M-H	C45H84NO10P	35.1	1.10	76.96
LIPIDS-NEG	2.24_279.2329m/z	0.003967	HMDB00673	Linoleic acid	M-H	C18H32O2	38.0	-0.36	90.56
MET-NEG	1.36_247.0623m/z	0.004378	HMDB29971	Coriandrone E	M-H	C13H12O5	37.4	4.63	92.32
LIPIDS-NEG	2.31_476.2774m/z	0.004893	123062684	PE(18:2(9Z,12Z)/0:0)	M-H	C23H44NO7P	38.0	-1.76	92.22
LIPIDS-NEG	2.24_504.3089m/z	0.005408	123061580	PC(17:2(9Z,12Z)/0:0)	M-H	C25H48NO7P	38.5	-1.29	93.99
LIPIDS-POS	6.23_772.5875m/z	0.007871	HMDB07946	PC(15:0/20:2(11Z,14Z))	M+H	C43H82NO8P	38.5	3.17	96.18
LIPIDS-POS	10.45_147.1171m/z	0.007878	HMDB61808	(3-Methyl-2-butenyl)-benzene	M+H	C11H14	38.4	2.16	94.37
MET-POS	0.70_205.1228m/z	0.008072	HMDB30770	4-Hydroxy-3-(3-methyl-2-butenyl)acetophenone	M+H	C13H16O2	39	2.660538241	98.33725638
LIPIDS-POS	5.48_715.5758m/z	0.009771	160780202	PE-Cer(d14:1(4E)/24:1(15Z))	M+H	C40H79N2O6P	38.4	1.31	93.38
LIPIDS-POS	5.31_622.4829m/z	0.010152	123062510	PE(O-16:0/12:0)	M+H	C33H68NO7P	37.7	3.63	92.70
LIPIDS-NEG	6.65_830.5929m/z	0.010338	123062910	PS(17:0/22:1(11Z))	M-H	C45H86NO10P	37.3	1.48	88.05
LIPIDS-POS	6.36_734.5732m/z	0.010499	HMDB00564	PC(16:0/16:0)	M+H	C40H80NO8P	38.7	5.15	99.30
MET-POS	4.69_520.3408m/z	0.011361	HMDB10386	LysoPC(18:2(9Z,12Z))	M+H	C26H50NO7P	35.4	2.024663355	79.28954476
LIPIDS-NEG	4.57_881.5204m/z	0.011440	123065375	PI(18:3(6Z,9Z,12Z)/20:3(8Z,11Z,14Z))	M-H	C47H79O13P	35.0	2.09	77.36
LIPIDS-POS	5.68_636.4975m/z	0.012283	HMDB29205	lysoPC(26:0)	M+H	C34H70NO7P	38.4	1.89	94.14
LIPIDS-POS	8.55_846.6971m/z	0.012972	HMDB07992	PC(16:0/24:0)	M+H	C48H96NO8P	38.0	2.87	93.51
LIPIDS-NEG	5.69_899.5677m/z	0.013032	123065234	PI(17:0/22:4(7Z,10Z,13Z,16Z))	M-H	C48H85O13P	37.4	2.49	90.15
LIPIDS-NEG	6.38_718.5390m/z	0.013489	HMDB07935	PC(15:0/16:0)	M-H	C39H78NO8P	35.9	-0.31	79.88
MET-POS	5.66_542.3215m/z	0.013587	HMDB10397	LysoPC(20:5(5Z,8Z,11Z,14Z,17Z))	M+H	C28H48NO7P	37.4	-4.903034111	92.78139033
LIPIDS-NEG	0.62_217.1076m/z	0.014020	HMDB00350	3-Hydroxysebacic acid	M-H	C10H18O5	38.3	-2.68	94.78
LIPIDS-NEG	6.10_768.5553m/z	0.014053	HMDB07947	PC(15:0/20:3(5Z,8Z,11Z))	M-H	C43H80NO8P	39.1	0.56	96.08
LIPIDS-NEG	6.02_816.5757m/z	0.014273	123062864	PS(16:0/22:1(11Z))	M-H	C44H84NO10P	35.3	-0.40	77.12
LIPIDS-NEG	5.21_871.5365m/z	0.014387	14714416	PI(17:0/20:4(5Z,8Z,11Z,14Z))	M-H	C46H81O13P	38.9	2.60	97.54
LIPIDS-POS	2.38_380.2567m/z	0.014448	HMDB00277	Sphingosine 1-phosphate	M+H	C18H38NO5P	38.8	1.65	96.14
LIPIDS-POS	5.79_923.5635m/z	0.014691	123065497	PI(19:1(9Z)/22:6(4Z,7Z,10Z,13Z,16Z,19Z))	M+H	C50H83O13P	38.8	-0.99	95.04
LIPIDS-POS	3.59_552.4054m/z	0.015104	HMDB10390	LysoPC(20:0)	M+H	C28H58NO7P	38.5	5.56	98.67
LIPIDS-NEG	2.24_578.3449m/z	0.016354	123063850	PS(22:1(11Z)/0:0)	M-H	C28H54NO9P	34.3	-2.50	74.33
LIPIDS-POS	6.68_748.5870m/z	0.016708	HMDB07937	PC(15:0/18:0)	M+H	C41H82NO8P	38.0	2.57	93.12
LIPIDS-POS	5.78_536.5054m/z	0.017691	123068733	Cer(d18:2/16:0)	M+H	C34H65NO3	37.9	3.17	93.46

LIPIDS-NEG	8.08_808.6233m/z	0.018178	123062577	PE(O-20:0/22:4(7Z,10Z,13Z,16Z))	M-H	C47H88NO7P	35.8	0.97	79.95
MET-POS	4.69_337.2740m/z	0.018982	HMDB06070	Pregnanetriol	M+H	C21H36O3	38.3	0.877453327	92.39690838
LIPIDS-NEG	5.81_899.5679m/z	0.019288	123065234	PI(17:0/22:4(7Z,10Z,13Z,16Z))	M-H	C48H85O13P	37.9	2.62	92.41
LIPIDS-POS	8.93_652.6617m/z	0.019627	7850636	Cer(d18:0/24:0)	M+H	C42H85NO3	39.1	2.21	98.23
LIPIDS-NEG	5.24_847.5356m/z	0.021005	123065077	PI(13:0/22:2(13Z,16Z))	M-H	C44H81O13P	37.7	1.67	90.69
MET-POS	2.41_282.2798m/z	0.021271	HMDB02117	Oleamide	M+H	C18H35NO	38.7	2.468121173	96.55601569
LIPIDS-NEG	6.02_802.5613m/z	0.021422	123062983	PS(18:0/19:1(9Z))	M-H	C43H82NO10P	38.5	1.14	93.94
MET-NEG	4.78_526.2967m/z	0.021987	HMDB11494	LysoPE(0:0/22:5(4Z,7Z,10Z,13Z,16Z))	M-H	C27H46NO7P	36.9	5.32	90.44
LIPIDS-POS	6.23_589.5198m/z	0.022409	LMFA07010691	Mayolene-20	M+H	C38H68O4	37.2	1.35	87.60
LIPIDS-POS	6.98_762.6048m/z	0.022457	HMDB07878	PC(14:0/20:0)	M+H	C42H84NO8P	38.4	5.38	98.31
LIPIDS-POS	3.51_552.4043m/z	0.022690	HMDB10390	LysoPC(20:0)	M+H	C28H58NO7P	38.6	3.50	96.94
LIPIDS-POS	8.55_383.3305m/z	0.022908	49703746	25-Dehydrovitamin D3	M+H	C27H42O	37.4	-0.91	88.30
MET-NEG	4.82_435.2506m/z	0.023551	HMDB07851	LPA(0:0/18:1(9Z))	M-H	C21H41O7P	38.5	-2.62	95.55
LIPIDS-POS	4.95_608.4676m/z	0.023950	HMDB10405	LysoPC(24:0)	M+H	C32H66NO7P	38.7	4.41	98.42
LIPIDS-NEG	0.57_1134.7871m/z	0.024172	HMDB04886	Trihexosylceramide (d18:1/24:0)	M-H	C60H113NO18	34.3	-1.20	72.99
LIPIDS-POS	5.63_520.3399m/z	0.024224	HMDB10386	LysoPC(18:2(9Z,12Z))	M+H	C26H50NO7P	38.0	0.35	90.29
MET-POS	0.81_135.0806m/z	0.024241	HMDB29697	Cinnamyl alcohol	M+H	C9H10O	38	1.515891307	91.82750097
MET-POS	4.39_703.5759m/z	0.024330	HMDB13464	SM(d18:0/16:1(9Z))	M+H	C39H79N2O6P	38.7	1.532680779	95.24213679
MET-POS	5.80_229.1557m/z	0.024893	HMDB11175	L-leucyl-L-proline	M+H	C11H20N2O3	38.3	4.455278644	96.73226447
MET-NEG	4.84_502.2929m/z	0.025245	HMDB11484	LysoPE(0:0/20:3(11Z,14Z,17Z))	M-H	C25H46NO7P	36.9	-2.08	87.17
LIPIDS-NEG	2.39_279.2328m/z	0.026677	HMDB00673	Linoleic acid	M-H	C18H32O2	37.2	-0.43	86.54
LIPIDS-NEG	7.53_802.5763m/z	0.026686	123062679	PE(P-20:0/22:6(4Z,7Z,10Z,13Z,16Z,19Z))	M-H	C47H82NO7P	35.7	0.87	79.67
LIPIDS-NEG	2.22_597.3040m/z	0.027055	74380468	PI(18:1(9Z)/0:0)	M-H	C27H51O12P	34.6	-0.86	74.29
MET-POS	2.04_266.0759m/z	0.027104	HMDB60663	Isoniazid alpha-ketoglutaric acid	M+H	C11H11N3O5	38.7	-<br>4.805090012	99.23963716
LIPIDS-NEG	6.45_675.4971m/z	0.027124	123067049	PA(18:0/16:0)	M-H	C37H73O8P	39.5	0.14	97.78
LIPIDS-POS	7.61_916.6780m/z	0.027519	HMDB08750	PC(22:6(4Z,7Z,10Z,13Z,16Z,19Z)/24:1(15Z))	M+H	C54H94NO8P	38.8	-1.05	95.15
LIPIDS-NEG	6.34_756.5550m/z	0.027564	HMDB07880	PC(14:0/20:2(11Z,14Z))	M-H	C42H80NO8P	35.6	0.16	78.30
LIPIDS-POS	5.78_770.5722m/z	0.027754	HMDB07947	PC(15:0/20:3(5Z,8Z,11Z))	M+H	C43H80NO8P	37.2	3.56	90.02
MET-NEG	0.73_187.1335m/z	0.027756	HMDB02203	3-Hydroxycapric acid	M-H	C10H20O3	38.4	-2.64	95.27
MET-NEG	4.86_466.2932m/z	0.028009	HMDB10379	LysoPC(14:0)	M-H	C22H46NO7P	38.9	-1.58	96.28
LIPIDS-NEG	8.11_799.6707m/z	0.028104	123068787	SM(d16:1/25:0)	M-H	C46H93N2O6P	37.9	1.00	90.74

LIPIDS-POS	7.26_776.6196m/z	0.028305	HMDB07944	PC(15:0/20:0)	M+H	C43H86NO8P	37.9	4.09	94.36
LIPIDS-POS	4.95_590.4554m/z	0.028395	160780188	CerP(d18:1/14:0)	M+H	C32H64NO6P	38.8	1.67	95.82
MET-NEG	3.71_476.2774m/z	0.029810	HMDB11477	LysoPE(0:0/18:2(9Z,12Z))	M-H	C23H44NO7P	34.4	-1.80	73.92
LIPIDS-POS	2.14_530.3261m/z	0.031105	HMDB11493	LysoPE(0:0/22:4(7Z,10Z,13Z,16Z))	M+H	C27H48NO7P	38.6	3.83	97.44
LIPIDS-NEG	0.69_187.0969m/z	0.031345	HMDB00784	Azelaic acid	M-H	C9H16O4	38.8	-3.62	98.09
MET-POS	4.77_504.3092m/z	0.031393	HMDB11484	LysoPE(0:0/20:3(11Z,14Z,17Z))	M+H	C25H46NO7P	38.1	1.38949082	92.11187596
LIPIDS-NEG	4.98_883.5366m/z	0.031550	HMDB09795	PI(16:0/22:5(4Z,7Z,10Z,13Z,16Z))	M-H	C47H81O13P	39.0	2.71	98.21
LIPIDS-POS	6.42_718.5761m/z	0.031785	HMDB07896	PC(14:0/P-18:0)	M+H	C40H80NO7P	37.7	2.19	91.32
MET-POS	0.85_177.0917m/z	0.032229	HMDB29699	Cinnamyl acetate	M+H	C11H12O2	37.6	3.924145804	92.41408869
MET-POS	6.07_203.1511m/z	0.032369	HMDB01539	Asymmetric dimethylarginine	M+H	C8H18N4O2	38.8	4.419259817	99.09016534
LIPIDS-POS	7.77_868.6799m/z	0.032879	HMDB08158	PC(18:2(9Z,12Z)/24:1(15Z))	M+H	C50H94NO8P	37.0	1.08	86.35
LIPIDS-POS	2.63_444.3701m/z	0.033386	HMDB13154	12-Hydroxy-12-octadecanoylcarnitine	M+H	C25H49NO5	38.4	4.01	96.77
MET-NEG	6.19_145.0971m/z	0.033400	HMDB00182	L-Lysine	M-H	C6H14N2O2	37.9	-7.97	98.22
LIPIDS-NEG	0.60_145.0976m/z	0.034013	HMDB00182	L-Lysine	M-H	C6H14N2O2	37.7	-4.76	93.92
LIPIDS-NEG	2.17_433.2352m/z	0.034157	123067332	PA(18:2(9Z,12Z)/0:0)	M-H	C21H39O7P	38.0	-2.05	92.25
LIPIDS-POS	8.28_870.6946m/z	0.034336	HMDB08092	PC(18:1(11Z)/24:1(15Z))	M+H	C50H96NO8P	37.4	-0.08	86.89
LIPIDS-POS	2.52_454.2934m/z	0.034850	HMDB11473	LysoPE(0:0/16:0)	M+H	C21H44NO7P	38.1	1.27	91.87
LIPIDS-POS	4.92_385.3454m/z	0.036179	HMDB00876	Vitamin D3	M+H	C27H44O	38.4	-2.89	95.57
LIPIDS-POS	5.50_554.5156m/z	0.036302	160780120	Cer(d14:1(4E)/20:0(2OH))	M+H	C34H67NO4	38.3	2.32	94.38
LIPIDS-NEG	1.90_619.2883m/z	0.036392	74380469	PI(20:4(5Z,8Z,11Z,14Z)/0:0)	M-H	C29H49O12P	34.8	-0.91	74.96
MET-NEG	6.19_203.0559m/z	0.037161	HMDB41031	3,5,6-Trihydroxy-5-(hydroxymethyl)-2-methoxy-2-cyclohexen-1-one	M-H	C8H12O6	38.0	-1.16	91.40
MET-NEG	0.73_187.0968m/z	0.038312	HMDB00784	Azelaic acid	M-H	C9H16O4	38.9	-4.35	99.42
LIPIDS-POS	2.48_382.2729m/z	0.038800	HMDB01383	Sphinganine 1-phosphate	M+H	C18H40NO5P	38.4	3.12	95.64
LIPIDS-NEG	2.78_494.3245m/z	0.039114	HMDB10382	LysoPC(16:0)	M-H	C24H50NO7P	37.7	-1.49	90.24
LIPIDS-NEG	2.31_466.2931m/z	0.039634	HMDB10379	LysoPC(14:0)	M-H	C22H46NO7P	35.8	-1.69	81.24
LIPIDS-POS	2.02_542.3272m/z	0.039908	HMDB10397	LysoPC(20:5(5Z,8Z,11Z,14Z,17Z))	M+H	C28H48NO7P	37.6	5.74	94.53
LIPIDS-NEG	4.10_474.3615m/z	0.039923	LMFA07070022	(7Z,10Z,13Z,16Z)-docosatetraenoylcarnitine	M-H	C29H49NO4	35.4	5.43	83.41
LIPIDS-POS	7.16_814.6364m/z	0.041531	HMDB07927	PC(14:1(9Z)/24:1(15Z))	M+H	C46H88NO8P	38.2	5.32	97.29
LIPIDS-POS	8.10_815.7036m/z	0.041632	HMDB12095	SM(d18:0/24:1(15Z))	M+H	C47H95N2O6P	38.7	4.39	98.81
LIPIDS-POS	3.22_576.4041m/z	0.041645	HMDB10400	LysoPC(22:2(13Z,16Z))	M+H	C30H58NO7P	38.9	2.94	98.07
LIPIDS-POS	1.51_427.2710m/z	0.041832	HMDB13043	Prostaglandin PGE2 1-glyceryl ester	M+H	C23H38O7	38.6	4.64	98.19

LIPIDS-POS	8.51_826.6682m/z	0.042126	HMDB08588	PC(22:1(13Z)/P-18:1(11Z))	M+H	C48H92NO7P	37.7	-0.25	88.65
LIPIDS-NEG	5.14_797.5223m/z	0.042651	123060581	MGDG(20:5(5Z,8Z,11Z,14Z,17Z)/18:3(9Z,12Z,15Z))	M-H	C47H74O10	34.7	1.70	75.77
MET-POS	1.30_342.1397m/z	0.042855	HMDB06591	Lactosamine	M+H	C12H23NO10	38.3	0.735096306	92.4065352
LIPIDS-POS	8.58_843.7325m/z	0.043476	7850653	SM(d18:1/26:0)	M+H	C49H99N2O6P	38.8	1.41	95.87
LIPIDS-POS	2.40_570.3584m/z	0.044179	HMDB10402	LysoPC(22:5(4Z,7Z,10Z,13Z,16Z))	M+H	C30H52NO7P	38.6	5.25	99.27
LIPIDS-POS	4.90_717.5557m/z	0.045649	HMDB13463	SM(d18:0/16:1(9Z)(OH))	M+H	C39H77N2O7P	38.9	2.27	97.36
LIPIDS-NEG	5.55_861.5520m/z	0.046684	HMDB09786	PI(16:0/20:2(11Z,14Z))	M-H	C45H83O13P	39.4	2.51	99.78
MET-NEG	1.80_512.2681m/z	0.047213	HMDB02639	Sulfolithocholylglycine	M-H	C26H43NO7S	36.4	-1.35	83.83
LIPIDS-POS	3.63_578.4201m/z	0.047327	HMDB10399	LysoPC(22:1(13Z))	M+H	C30H60NO7P	38.9	3.57	98.50
LIPIDS-NEG	2.13_311.2223m/z	0.048450	HMDB03871	13-L-Hydroperoxylinoleic acid	M-H	C18H32O4	36.5	-1.45	84.05
MET-NEG	5.24_195.0497m/z	0.049080	HMDB00565	Galactonic acid	M-H	C6H12O7	36.4	-6.98	89.79
LIPIDS-POS	4.25_562.4250m/z	0.049133	HMDB10699	CerP(d18:1/12:0)	M+H	C30H60NO6P	38.4	3.46	96.13
LIPIDS-POS	5.68_561.4897m/z	0.049338	123060444	Mayolene-18	M+H	C36H64O4	38.1	3.53	94.81
LIPIDS-POS	3.35_564.4042m/z	0.049445	HMDB11498	LysoPE(0:0/24:1(15Z))	M+H	C29H58NO7P	37.3	3.32	90.27
LIPIDS-POS	2.13_445.2954m/z	0.049648	HMDB00969	1,25-Dihydroxyvitamin D3-26,23-lactone	M+H	C27H40O5	38.6	1.15	94.21
LIPIDS-NEG	7.48_778.5760m/z	0.049869	HMDB09610	PE(22:4(7Z,10Z,13Z,16Z)/P-18:0)	M-H	C45H82NO7P	35.4	0.50	77.78
MET-POS	6.36_156.0772m/z	0.049895	HMDB00177	L-Histidine	M+H	C6H9N3O2	39	2.599843993	97.95242006

Day 21 post-exposure

Method	Compounds	ANOVA p-value	Accepted Compound ID	Accepted Description	Adducts	Formula	Score	Mass Error (ppm)	Isotope Similarity
LIPIDS-NEG	6.45_675.4971m/z	0.000101	123067049	PA(18:0/16:0)	M-H	C37H73O8P	39.5	0.14	97.78
LIPIDS-POS	2.52_454.2934m/z	0.001001	HMDB11473	LysoPE(0:0/16:0)	M+H	C21H44NO7P	38.1	1.27	91.87
LIPIDS-NEG	5.86_790.5398m/z	0.001483	HMDB07958	PC(15:0/22:6(4Z,7Z,10Z,13Z,16Z,19Z))	M-H	C45H78NO8P	38.7	0.77	94.46
LIPIDS-NEG	6.02_816.5757m/z	0.001685	123062864	PS(16:0/22:1(11Z))	M-H	C44H84NO10P	35.3	-0.40	77.12
MET-POS	3.58_341.3050m/z	0.001718	7982761	2-oxo-heneicosanoic acid	M+H	C21H40O3	37.2	0.00	86.13
MET-NEG	1.28_206.0811m/z	0.001763	HMDB00860	Phenylpropionylglycine	M-H	C11H13NO3	35.6	-5.56	84.61
LIPIDS-NEG	5.13_859.5360m/z	0.002010	HMDB09787	PI(16:0/20:3(5Z,8Z,11Z))	M-H	C45H81O13P	39.0	2.07	97.28
LIPIDS-NEG	6.02_802.5613m/z	0.002238	123062983	PS(18:0/19:1(9Z))	M-H	C43H82NO10P	38.5	1.14	93.94

LIPIDS-NEG	2.55_255.2328m/z	0.002299	HMDB00220	Palmitic acid	M-H	C16H32O2	38.4	-0.67	92.56
LIPIDS-NEG	2.62_452.2777m/z	0.002514	HMDB11473	LysoPE(0:0/16:0)	M-H	C21H44NO7P	39.5	-1.21	98.88
LIPIDS-NEG	6.65_790.5399m/z	0.002528	HMDB07958	PC(15:0/22:6(4Z,7Z,10Z,13Z,16Z,19Z))	M-H	C45H78NO8P	37.0	0.88	86.19
MET-POS	4.69_337.2740m/z	0.003134	HMDB06070	Pregnanetriol	M+H	C21H36O3	38.3	0.88	92.40
MET-NEG	3.99_718.5384m/z	0.003151	HMDB07935	PC(15:0/16:0)	M-H	C39H78NO8P	37.5	-1.16	89.04
MET-POS	3.71_160.0763m/z	0.003209	HMDB01190	Indoleacetaldehyde	M+H	C10H9NO	37.4	3.65	91.40
LIPIDS-POS	5.79_385.3461m/z	0.003275	HMDB00876	Vitamin D3	M+H	C27H44O	38.1	-1.01	91.71
LIPIDS-NEG	6.22_764.5240m/z	0.003375	HMDB07951	PC(15:0/20:5(5Z,8Z,11Z,14Z,17Z))	M-H	C43H76NO8P	38.2	0.49	91.57
LIPIDS-NEG	6.14_738.5083m/z	0.003579	HMDB07943	PC(15:0/18:4(6Z,9Z,12Z,15Z))	M-H	C41H74NO8P	38.9	0.46	95.00
LIPIDS-POS	2.26_454.3312m/z	0.003934	14713822	PC(O-12:0/O-2:0)	M+H	C22H48NO6P	38.3	4.30	96.33
LIPIDS-NEG	5.85_875.5672m/z	0.003972	123065320	PI(18:1(9Z)/19:1(9Z))	M-H	C46H85O13P	35.4	1.98	79.36
MET-NEG	6.43_195.0499m/z	0.004033	HMDB00565	Galactonic acid	M-H	C6H12O7	37.6	-5.78	94.40
LIPIDS-NEG	4.93_833.5202m/z	0.004060	HMDB09784	PI(16:0/18:2(9Z,12Z))	M-H	C43H79O13P	39.3	1.97	99.04
LIPIDS-NEG	6.01_762.5083m/z	0.004485	HMDB08946	PE(16:0/22:6(4Z,7Z,10Z,13Z,16Z,19Z))	M-H	C43H74NO8P	37.5	0.44	87.89
LIPIDS-NEG	2.53_452.2774m/z	0.004859	HMDB11473	LysoPE(0:0/16:0)	M-H	C21H44NO7P	38.2	-1.80	93.11
LIPIDS-NEG	6.77_766.5400m/z	0.005206	HMDB07949	PC(15:0/20:4(5Z,8Z,11Z,14Z))	M-H	C43H78NO8P	39.4	1.02	98.05
LIPIDS-POS	2.61_454.2935m/z	0.005786	HMDB11473	LysoPE(0:0/16:0)	M+H	C21H44NO7P	39.0	1.51	96.62
LIPIDS-POS	10.46_149.1327m/z	0.005958	HMDB59834	Pentylbenzene	M+H	C11H16	38.6	1.75	94.92
MET-NEG	0.75_255.1593m/z	0.006565	HMDB36143	Monomenthyl succinate	M-H	C14H24O4	37.7	-3.63	92.84
LIPIDS-NEG	6.21_768.5550m/z	0.006996	HMDB07947	PC(15:0/20:3(5Z,8Z,11Z))	M-H	C43H80NO8P	37.3	0.15	86.67
LIPIDS-NEG	5.97_826.5616m/z	0.007947	123062940	PS(17:1(9Z)/22:2(13Z,16Z))	M-H	C45H82NO10P	37.9	1.50	91.14
LIPIDS-POS	2.69_540.3674m/z	0.008113	123063861	PS(O-20:0/0:0)	M+H	C26H54NO8P	39.1	2.60	98.80
LIPIDS-NEG	6.48_804.5770m/z	0.008845	123062821	PS(15:0/22:0)	M-H	C43H84NO10P	33.7	1.17	69.96
LIPIDS-POS	10.73_673.5911m/z	0.010824	HMDB06726	CE(20:4(5Z,8Z,11Z,14Z)	M+H	C47H76O2	39.1	-1.04	96.79
MET-NEG	2.47_885.5513m/z	0.010849	HMDB09793	PI(16:0/22:4(10Z,13Z,16Z,19Z))	M-H	C47H83O13P	38.7	1.67	95.58
LIPIDS-POS	6.76_768.5562m/z	0.011102	HMDB07949	PC(15:0/20:4(5Z,8Z,11Z,14Z))	M+H	C43H78NO8P	37.5	3.18	91.05
LIPIDS-NEG	5.97_766.5399m/z	0.011546	HMDB07949	PC(15:0/20:4(5Z,8Z,11Z,14Z))	M-H	C43H78NO8P	39.5	0.93	98.48
MET-NEG	3.80_818.5710m/z	0.011824	HMDB09243	PE(20:0/22:6(4Z,7Z,10Z,13Z,16Z,19Z))	M-H	C47H82NO8P	36.6	0.54	83.50
LIPIDS-POS	2.61_433.2350m/z	0.011977	123067338	PA(18:3(6Z,9Z,12Z)/0:0)	M+H	C21H37O7P	38.9	0.00	94.43
MET-NEG	1.73_498.2882m/z	0.012106	HMDB00896	Taurodeoxycholic acid	M-H	C26H45NO6S	37.8	-2.62	92.19
MET-NEG	2.81_766.5389m/z	0.012288	HMDB09228	PE(20:0/18:4(6Z,9Z,12Z,15Z))	M-H	C43H78NO8P	36.8	-0.44	84.57
MET-NEG	0.69_163.0388m/z	0.012427	HMDB00205	Phenylpyruvic acid	M-H	C9H8O3	37.0	-7.52	93.57

MET-NEG	0.72_215.1643m/z	0.012589	HMDB00387	3-Hydroxydodecanoic acid	M-H	C12H24O3	38.1	-4.26	95.54
MET-NEG	3.85_303.2320m/z	0.012679	HMDB01043	Arachidonic acid	M-H	C20H32O2	35.8	-3.22	82.78
LIPIDS-POS	7.29_681.4894m/z	0.012837	123067217	PA(O-16:0/20:5(5Z,8Z,11Z,14Z,17Z))	M+H	C39H69O7P	37.4	5.99	93.90
MET-NEG	1.19_245.0928m/z	0.013259	HMDB13713	N-acetyltryptophan	M-H	C13H14N2O3	37.9	-1.48	91.05
LIPIDS-POS	2.38_380.2567m/z	0.013669	HMDB00277	Sphingosine 1-phosphate	M+H	C18H38NO5P	38.8	1.65	96.14
MET-POS	1.17_275.1465m/z	0.013859	HMDB28824	Glutamyl-Lysine	M+H	C11H20N3O5-	37.8	-3.96	93.66
LIPIDS-NEG	4.80_881.5206m/z	0.015498	123065030	PI(16:0/22:6(4Z,7Z,10Z,13Z,16Z,19Z))	M-H	C47H79O13P	38.4	2.36	95.01
LIPIDS-NEG	2.31_524.2778m/z	0.015723	HMDB11496	LysoPE(0:0/22:6(4Z,7Z,10Z,13Z,16Z,19Z))	M-H	C27H44NO7P	38.5	-0.94	93.48
LIPIDS-POS	5.12_883.5327m/z	0.016260	123065030	PI(16:0/22:6(4Z,7Z,10Z,13Z,16Z,19Z))	M+H	C47H79O13P	39.4	-0.43	97.48
MET-POS	3.72_834.5997m/z	0.016324	HMDB08057	PC(18:0/22:6(4Z,7Z,10Z,13Z,16Z,19Z))	M+H	C48H84NO8P	35.9	-1.20	81.01
MET-POS	3.92_706.5391m/z	0.016658	HMDB07869	PC(14:0/16:0)	M+H	C38H76NO8P	38.2	1.32	92.79
LIPIDS-POS	0.99_427.1863m/z	0.016896	HMDB30378	(Z)-Narceine imide	M+H	C23H26N2O6	37.8	-0.11	88.98
MET-POS	6.36_156.0772m/z	0.016990	HMDB00177	L-Histidine	M+H	C6H9N3O2	39.0	2.60	97.95
LIPIDS-NEG	4.90_857.5204m/z	0.017113	HMDB09789	PI(16:0/20:4(5Z,8Z,11Z,14Z))	M-H	C45H79O13P	39.3	2.21	99.29
LIPIDS-NEG	0.62_498.2878m/z	0.017186	HMDB00874	Tauroursodeoxycholic acid	M-H	C26H45NO6S	37.7	-3.40	92.49
MET-POS	1.21_112.0503m/z	0.017478	HMDB00630	Cytosine	M+H	C4H5N3O	39.3	-1.94	98.72
LIPIDS-POS	2.97_494.3620m/z	0.017652	14713968	PC(P-17:0/0:0)	M+H	C25H52NO6P	38.2	3.05	94.40
LIPIDS-NEG	5.35_835.5356m/z	0.018008	4266294	PI(16:0/18:1(9Z))	M-H	C43H81O13P	38.7	1.72	95.47
LIPIDS-POS	2.75_564.3682m/z	0.018233	7983365	PC(18:1(9E)/2:0)	M+H	C28H54NO8P	38.6	3.98	97.77
LIPIDS-NEG	3.10_534.3559m/z	0.018291	123061583	PC(19:1(9Z)/0:0)	M-H	C27H54NO7P	34.7	-1.14	74.65
LIPIDS-POS	2.28_380.2565m/z	0.018380	HMDB00277	Sphingosine 1-phosphate	M+H	C18H38NO5P	39.1	1.11	97.02
MET-NEG	3.83_794.5706m/z	0.018454	HMDB07955	PC(15:0/22:4(7Z,10Z,13Z,16Z))	M-H	C45H82NO8P	38.4	0.15	92.11
MET-NEG	0.76_189.0756m/z	0.018588	HMDB00325	3-Hydroxysuberic acid	M-H	C8H14O5	36.9	-6.50	91.93
MET-NEG	3.72_452.2774m/z	0.019564	HMDB11473	LysoPE(0:0/16:0)	M-H	C21H44NO7P	38.2	-1.90	93.21
LIPIDS-POS	5.12_717.5557m/z	0.020195	HMDB13463	SM(d18:0/16:1(9Z)(OH))	M+H	C39H77N2O7P	37.7	2.24	91.32
MET-NEG	0.71_413.2182m/z	0.020274	HMDB30895	(4R,5S,7R,11x)-11,12-Dihydroxy-1(10)-spirovetiven-2-one 12-glucoside	M-H	C21H34O8	38.8	0.19	94.00
LIPIDS-NEG	6.47_744.5551m/z	0.020453	HMDB07938	PC(15:0/18:1(11Z))	M-H	C41H80NO8P	38.7	0.23	93.67
MET-POS	3.62_454.2925m/z	0.020456	HMDB11473	LysoPE(0:0/16:0)	M+H	C21H44NO7P	39.0	-0.72	96.07
MET-NEG	2.53_188.0359m/z	0.021232	HMDB00715	Kynurenic acid	M-H	C10H7NO3	38.3	3.11	95.40
LIPIDS-POS	7.51_854.6621m/z	0.021263	HMDB09311	PE(20:2(11Z,14Z)/24:1(15Z))	M+H	C49H92NO8P	36.7	-1.41	85.35
LIPIDS-NEG	6.60_854.5932m/z	0.021680	123063173	PS(19:1(9Z)/22:2(13Z,16Z))	M-H	C47H86NO10P	37.7	1.75	90.80

MET-POS	3.54_526.2904m/z	0.021733	HMDB11496	LysoPE(0:0/22:6(4Z,7Z,10Z,13Z,16Z,19Z))	M+H	C27H44NO7P	37.5	-4.69	92.69
MET-NEG	3.94_279.2321m/z	0.022022	HMDB00673	Linoleic acid	M-H	C18H32O2	36.3	-3.22	85.39
LIPIDS-POS	3.51_552.4043m/z	0.022114	HMDB10390	LysoPC(20:0)	M+H	C28H58NO7P	38.6	3.50	96.94
MET-NEG	2.49_857.5199m/z	0.022597	HMDB09789	PI(16:0/20:4(5Z,8Z,11Z,14Z))	M-H	C45H79O13P	36.6	1.60	84.71
LIPIDS-POS	10.45_369.3530m/z	0.023042	49703700	3-Deoxyvitamin D3	M+H	C27H44	38.9	3.91	99.27
LIPIDS-POS	5.83_828.5539m/z	0.023603	HMDB08189	PC(18:3(6Z,9Z,12Z)/22:6(4Z,7Z,10Z,13Z,16Z,19Z))	M+H	C48H78NO8P	37.3	0.10	86.45
MET-NEG	6.40_154.0610m/z	0.023837	HMDB00177	L-Histidine	M-H	C6H9N3O2	37.3	-7.86	95.47
LIPIDS-POS	3.35_564.4042m/z	0.024109	HMDB11498	LysoPE(0:0/24:1(15Z))	M+H	C29H58NO7P	37.3	3.32	90.27
LIPIDS-NEG	5.24_847.5356m/z	0.024500	123065077	PI(13:0/22:2(13Z,16Z))	M-H	C44H81O13P	37.7	1.67	90.69
MET-NEG	1.26_284.0878m/z	0.024675	HMDB05923	N4-Acetylcytidine	M-H	C11H15N3O6	38.2	-3.60	95.25
MET-POS	3.63_476.2748m/z	0.024736	HMDB11478	LysoPE(0:0/18:3(6Z,9Z,12Z))	M+H	C23H42NO7P	37.6	-5.03	93.64
MET-NEG	3.84_790.5397m/z	0.024890	HMDB07958	PC(15:0/22:6(4Z,7Z,10Z,13Z,16Z,19Z))	M-H	C45H78NO8P	32.4	0.65	62.57
LIPIDS-NEG	0.89_464.3007m/z	0.025335	HMDB00138	Glycocholic acid	M-H	C26H43NO6	37.1	-2.37	88.34
LIPIDS-POS	10.73_369.3529m/z	0.025390	49703700	3-Deoxyvitamin D3	M+H	C27H44	38.9	3.70	99.07
MET-NEG	3.87_766.5395m/z	0.025470	HMDB07949	PC(15:0/20:4(5Z,8Z,11Z,14Z))	M-H	C43H78NO8P	34.9	0.30	75.02
MET-POS	3.93_728.5208m/z	0.026004	HMDB07875	PC(14:0/18:3(6Z,9Z,12Z))	M+H	C40H74NO8P	37.5	-2.36	90.48
LIPIDS-NEG	7.60_804.5918m/z	0.026413	123062578	PE(O-20:0/22:6(4Z,7Z,10Z,13Z,16Z,19Z))	M-H	C47H84NO7P	36.3	0.66	82.49
LIPIDS-NEG	6.48_818.5727m/z	0.026707	HMDB09243	PE(20:0/22:6(4Z,7Z,10Z,13Z,16Z,19Z))	M-H	C47H82NO8P	38.5	2.71	95.94
MET-NEG	4.61_195.0500m/z	0.027702	HMDB00565	Galactonic acid	M-H	C6H12O7	36.3	-5.18	87.41
LIPIDS-NEG	6.38_718.5390m/z	0.028038	HMDB07935	PC(15:0/16:0)	M-H	C39H78NO8P	35.9	-0.31	79.88
LIPIDS-POS	0.88_455.2388m/z	0.028058	123065022	PG(14:1(9Z)/0:0)	M+H	C20H39O9P	37.9	-3.63	93.75
MET-NEG	1.92_514.2837m/z	0.028306	HMDB00036	Taurocholic acid	M-H	C26H45NO7S	37.9	-1.44	91.39
LIPIDS-NEG	0.52_273.0012m/z	0.028766	HMDB03976	D-Glucuronic acid 1-phosphate	M-H	C6H11O10P	37.0	-2.01	87.59
MET-NEG	0.73_187.1335m/z	0.028790	HMDB02203	3-Hydroxycapric acid	M-H	C10H20O3	38.4	-2.64	95.27
LIPIDS-NEG	6.02_742.5398m/z	0.029046	HMDB07940	PC(15:0/18:2(9Z,12Z))	M-H	C41H78NO8P	39.6	0.78	98.91
LIPIDS-NEG	5.81_899.5679m/z	0.029634	123065234	PI(17:0/22:4(7Z,10Z,13Z,16Z))	M-H	C48H85O13P	37.9	2.62	92.41
MET-NEG	3.80_878.5924m/z	0.029749	HMDB61473	PE(DiMe(11,3)/DiMe(13,5))	M-H	C49H86NO10P	37.8	0.82	90.17
LIPIDS-NEG	6.65_830.5929m/z	0.029834	123062910	PS(17:0/22:1(11Z))	M-H	C45H86NO10P	37.3	1.48	88.05
LIPIDS-POS	1.51_469.3147m/z	0.030541	14715153	(6RS)-6,19-epidioxy-24,24-difluoro-25-hydroxy-6,19-dihydrovitamin D3 / (6RS)-6,19-epidioxy-24,24-difluoro-25-hydroxy-6,19-dihydrocholecalciferol	M+H	C27H42F2O4	38.7	4.87	99.35
LIPIDS-POS	4.93_879.4985m/z	0.031868	123065377	PI(18:3(6Z,9Z,12Z)/20:5(5Z,8Z,11Z,14Z,17Z))	M+H	C47H75O13P	35.8	-3.72	83.57

MET-POS	5.68_112.0868m/z	0.031917	HMDB00870	Histamine	M+H	C5H9N3	38.8	-0.92	94.91
LIPIDS-NEG	6.38_764.5238m/z	0.032144	HMDB07951	PC(15:0/20:5(5Z,8Z,11Z,14Z,17Z))	M-H	C43H76NO8P	35.2	0.34	76.49
MET-NEG	2.51_833.5195m/z	0.032164	HMDB09784	PI(16:0/18:2(9Z,12Z))	M-H	C43H79O13P	38.8	1.08	95.18
LIPIDS-NEG	0.90_273.1705m/z	0.032230	HMDB00394	3-Hydroxytetradecanedioic acid	M-H	C14H26O5	39.3	-0.94	97.85
LIPIDS-NEG	3.01_480.3089m/z	0.033758	7983912	PC(15:0/0:0)	M-H	C23H48NO7P	38.7	-1.39	95.29
LIPIDS-NEG	5.65_849.5512m/z	0.034585	123065076	PI(13:0/22:1(11Z))	M-H	C44H83O13P	31.6	1.64	59.81
LIPIDS-POS	6.75_790.5379m/z	0.034823	HMDB09045	PE(18:1(11Z)/22:6(4Z,7Z,10Z,13Z,16Z,19Z))	M+H	C45H76NO8P	39.2	-0.25	96.38
MET-POS	4.42_496.3398m/z	0.035448	HMDB10382	LysoPC(16:0)	M+H	C24H50NO7P	38.5	-0.01	92.59
LIPIDS-POS	1.35_413.2549m/z	0.035526	HMDB03752	LysoPC(10:0)	M+H	C18H39NO7P+	36.8	2.85	87.43
LIPIDS-POS	10.46_671.5749m/z	0.035738	HMDB06731	CE(20:5(5Z,8Z,11Z,14Z,17Z)	M+H	C47H74O2	39.1	-1.87	97.58
LIPIDS-NEG	0.85_498.2885m/z	0.036589	HMDB00874	Tauroursodeoxycholic acid	M-H	C26H45NO6S	38.2	-1.93	93.44
LIPIDS-NEG	2.03_325.1839m/z	0.037190	HMDB31031	2-Dodecylbenzenesulfonic acid	M-H	C18H30O3S	35.6	-1.33	79.83
LIPIDS-POS	5.82_806.5734m/z	0.037539	HMDB07991	PC(16:0/22:6(4Z,7Z,10Z,13Z,16Z,19Z))	M+H	C46H80NO8P	37.3	4.88	91.96
LIPIDS-POS	10.73_147.1171m/z	0.037582	HMDB61808	(3-Methyl-2-butenyl)-benzene	M+H	C11H14	38.2	2.19	93.42
LIPIDS-NEG	5.55_861.5520m/z	0.037902	HMDB09786	PI(16:0/20:2(11Z,14Z))	M-H	C45H83O13P	39.4	2.51	99.78
MET-POS	4.57_480.3446m/z	0.038403	HMDB10407	LysoPC(P-16:0)	M+H	C24H50NO6P	39.1	-0.42	95.85
LIPIDS-POS	6.13_740.5239m/z	0.038750	HMDB07943	PC(15:0/18:4(6Z,9Z,12Z,15Z))	M+H	C41H74NO8P	37.6	1.95	90.15
MET-POS	0.72_231.0985m/z	0.038759	HMDB02335	Aspartyl-L-proline	M+H	C9H14N2O5	38.3	4.01	96.39
MET-POS	3.85_98.9840m/z	0.039150	HMDB02142	Phosphoric acid	M+H	H3O4P	39.3	-2.21	99.00
MET-NEG	3.95_742.5394m/z	0.039402	HMDB07940	PC(15:0/18:2(9Z,12Z))	M-H	C41H78NO8P	38.1	0.24	90.59
MET-POS	3.86_758.5706m/z	0.039811	HMDB07880	PC(14:0/20:2(11Z,14Z))	M+H	C42H80NO8P	37.6	1.56	90.04
MET-POS	3.57_482.3237m/z	0.040550	HMDB10381	LysoPC(15:0)	M+H	C23H48NO7P	38.8	-0.85	95.18
MET-POS	0.65_281.1022m/z	0.041188	HMDB33884	Gravolenic acid	M+H	C14H16O6	39.2	0.88	97.22
MET-NEG	0.72_185.1172m/z	0.042286	HMDB10724	3-Oxodecanoic acid	M-H	C10H18O3	38.2	-5.90	97.63
LIPIDS-NEG	6.60_794.5716m/z	0.042838	HMDB07955	PC(15:0/22:4(7Z,10Z,13Z,16Z))	M-H	C45H82NO8P	39.3	1.38	98.12
LIPIDS-NEG	5.49_883.5361m/z	0.043486	HMDB09795	PI(16:0/22:5(4Z,7Z,10Z,13Z,16Z))	M-H	C47H81O13P	34.9	2.14	76.93
LIPIDS-POS	5.49_804.5561m/z	0.045584	HMDB08023	PC(16:1(9Z)/22:6(4Z,7Z,10Z,13Z,16Z,19Z))	M+H	C46H78NO8P	37.1	2.87	88.93
LIPIDS-NEG	5.96_863.5673m/z	0.045636	123065093	PI(14:0/22:1(11Z))	M-H	C45H85O13P	38.5	2.09	95.22
MET-NEG	0.69_465.2463m/z	0.045674	HMDB02829	Androsterone glucuronide	M-H	C25H38O8	37.8	-6.73	96.78
LIPIDS-NEG	7.02_776.5602m/z	0.046864	HMDB09611	PE(22:4(7Z,10Z,13Z,16Z)/P-18:1(11Z))	M-H	C45H80NO7P	37.8	0.36	89.38
MET-POS	2.16_126.0663m/z	0.047081	HMDB02894	5-Methylcytosine	M+H	C5H7N3O	39.5	1.00	98.53
LIPIDS-NEG	1.50_443.2641m/z	0.047515	49703495	1(3)-glyceryl-6-keto-PGF1alpha	M-H	C23H40O8	37.7	-2.14	91.16

LIPIDS-NEG	7.55_780.5915m/z	0.047763	HMDB09578	PE(22:2(13Z,16Z)/P-18:1(11Z))	M-H	C45H84NO7P	36.8	0.33	84.62
LIPIDS-POS	10.45_147.1171m/z	0.048197	HMDB61808	(3-Methyl-2-butenyl)-benzene	M+H	C11H14	38.4	2.16	94.37
LIPIDS-NEG	5.51_885.5526m/z	0.048255	HMDB09793	PI(16:0/22:4(10Z,13Z,16Z,19Z))	M-H	C47H83O13P	39.2	3.05	99.72
MET-NEG	2.50_861.5500m/z	0.048642	HMDB09786	PI(16:0/20:2(11Z,14Z))	M-H	C45H83O13P	37.8	0.13	89.36
LIPIDS-NEG	6.17_818.5714m/z	0.049388	HMDB09243	PE(20:0/22:6(4Z,7Z,10Z,13Z,16Z,19Z))	M-H	C47H82NO8P	33.8	1.03	70.40
LIPIDS-NEG	6.65_770.5713m/z	0.049528	HMDB07946	PC(15:0/20:2(11Z,14Z))	M-H	C43H82NO8P	39.2	0.97	97.23
MET-NEG	0.70_313.2371m/z	0.049714	HMDB00782	Octadecanedioic acid	M-H	C18H34O4	37.3	-4.10	91.07

Table S5

Pathway analysis were carried out using MetaboAnalyst software using Hypergeometric Test for over-representative analysis and the Relative-betweeness Centrality for pathway topology with *Rattus norvegicus* pathway library. Pathway that are significant are indicated in bold.

Day 1 post-exposure

	**Total**	**Expected**	**Hits**	**p-value**	**-LOG(p)**	**Impact**
Glycerophospholipid metabolism	30	0.44936	2	0.072277	2.6273	0.18333
Linoleic acid metabolism	5	0.074893	1	0.072784	2.6203	0
Biotin metabolism	5	0.074893	1	0.072784	2.6203	0
Pyrimidine metabolism	41	0.61412	2	0.12356	2.091	0.05384
alpha-Linolenic acid metabolism	9	0.13481	1	0.12735	2.0608	0
Nitrogen metabolism	9	0.13481	1	0.12735	2.0608	0
Pantothenate and CoA biosynthesis	15	0.22468	1	0.2035	1.5921	0
Histidine metabolism	15	0.22468	1	0.2035	1.5921	0.24194
Glyoxylate and dicarboxylate metabolism	16	0.23966	1	0.21556	1.5345	0.2963
beta-Alanine metabolism	19	0.28459	1	0.2507	1.3835	0
Citrate cycle (TCA cycle)	20	0.29957	1	0.26208	1.3391	0.05356
Aminoacyl-tRNA biosynthesis	67	1.0036	2	0.26538	1.3266	0
Porphyrin and chlorophyll metabolism	27	0.40442	1	0.33724	1.087	0.0415
Arachidonic acid metabolism	36	0.53923	1	0.4232	0.85991	0
Primary bile acid biosynthesis	46	0.68902	1	0.50624	0.68075	0
Purine metabolism	68	1.0185	1	0.65069	0.42973	0.02077
Steroid hormone biosynthesis	70	1.0485	1	0.6616	0.41309	0.02548

Day 5 post-exposure

	**Total**	**Expected**	**Hits**	**p-value**	**-LOG(p)**	**Impact**
**Biosynthesis of unsaturated fatty acids**	**42**	**0.59914**	**7**	**7.53E-07**	**14.1**	**0**

**Linoleic acid metabolism**	**5**	**0.071327**	**2**	**0.0018853**	**6.2737**	**1**
**alpha-Linolenic acid metabolism**	**9**	**0.12839**	**2**	**0.006558**	**5.0271**	**1**
Phenylalanine, tyrosine and tryptophan biosynthesis	4	0.057061	1	0.05591	2.884	0.5
Glycerophospholipid metabolism	30	0.42796	2	0.066257	2.7142	0.18333
Arachidonic acid metabolism	36	0.51355	2	0.091216	2.3945	0.32601
Nitrogen metabolism	9	0.12839	1	0.12163	2.1068	0
Phenylalanine metabolism	9	0.12839	1	0.12163	2.1068	0.40741
Nicotinate and nicotinamide metabolism	13	0.18545	1	0.17105	1.7658	0.2381
Histidine metabolism	15	0.21398	1	0.19476	1.636	0.24194
Aminoacyl-tRNA biosynthesis	67	0.95578	2	0.24724	1.3974	0
Sphingolipid metabolism	21	0.29957	1	0.26208	1.3391	0.03008
Steroid biosynthesis	35	0.49929	1	0.39898	0.91884	0
Tryptophan metabolism	41	0.58488	1	0.44994	0.79865	0
Fatty acid biosynthesis	43	0.61341	1	0.46599	0.76359	0
Steroid hormone biosynthesis	70	0.99857	1	0.64355	0.44076	0

Day 9 post-exposure

	Total	Expected	Hits	p-value	-LOG(p)	Impact
**Biosynthesis of unsaturated fatty acids**	**42**	**0.92867**	**4**	**0.012172**	**4.4086**	**0**
**Glycerophospholipid metabolism**	**30**	**0.66334**	**3**	**0.02654**	**3.6291**	**0.20648**
Retinol metabolism	17	0.37589	2	0.052366	2.9495	0.47938
Tryptophan metabolism	41	0.90656	3	0.059173	2.8273	0.1863
Linoleic acid metabolism	5	0.11056	1	0.10592	2.2451	0
alpha-Linolenic acid metabolism	9	0.199	1	0.18276	1.6996	0
Fatty acid metabolism	39	0.86234	2	0.21233	1.5496	0
Fatty acid biosynthesis	43	0.95078	2	0.24548	1.4045	0
Primary bile acid biosynthesis	46	1.0171	2	0.27053	1.3074	0.01057
Pantothenate and CoA biosynthesis	15	0.33167	1	0.28616	1.2512	0.02041
Glyoxylate and dicarboxylate metabolism	16	0.35378	1	0.30211	1.1969	0.2963
Citrate cycle (TCA cycle)	20	0.44223	1	0.36255	1.0146	0.05356

Sphingolipid metabolism	21	0.46434	1	0.37685	0.97591	0.2807
Fatty acid elongation in mitochondria	27	0.597	1	0.45634	0.78451	0
Porphyrin and chlorophyll metabolism	27	0.597	1	0.45634	0.78451	0.0415
Arachidonic acid metabolism	36	0.79601	1	0.55748	0.58432	0
Pyrimidine metabolism	41	0.90656	1	0.60553	0.50164	0.01202
Aminoacyl-tRNA biosynthesis	67	1.4815	1	0.78453	0.24267	0

Day 13 post-exposure

	Total	Expected	Hits	p-value	-LOG(p)	Impact
**Glycerophospholipid metabolism**	**30**	**0.27817**	**3**	**0.0021904**	**6.1237**	**0.25525**
**Linoleic acid metabolism**	**5**	**0.046362**	**1**	**0.045574**	**3.0884**	**0**
alpha-Linolenic acid metabolism	9	0.083452	1	0.080645	2.5177	0
Glycosylphosphatidylinositol(GPI)-anchor biosynthesis	14	0.12981	1	0.12281	2.0971	0.0439
Sphingolipid metabolism	21	0.19472	1	0.17885	1.7212	0.03008
Steroid biosynthesis	35	0.32454	1	0.28114	1.2689	0
Arachidonic acid metabolism	36	0.33381	1	0.28798	1.2449	0
Arginine and proline metabolism	44	0.40799	1	0.34054	1.0772	0

Day 17 post-exposure

	Total	Expected	Hits	p-value	-LOG(p)	Impact
**Linoleic acid metabolism**	**5**	**0.082026**	**3**	**3.78E-05**	**10.183**	**1**
**Glycerophospholipid metabolism**	**30**	**0.49215**	**4**	**0.0011388**	**6.7777**	**0.47253**
**Sphingolipid metabolism**	**21**	**0.34451**	**3**	**0.0042341**	**5.4646**	**0.05264**
Biotin metabolism	5	0.082026	1	0.079488	2.5322	0
alpha-Linolenic acid metabolism	9	0.14765	1	0.13869	1.9755	0
Nitrogen metabolism	9	0.14765	1	0.13869	1.9755	0
Glycosylphosphatidylinositol(GPI)-anchor biosynthesis	14	0.22967	1	0.20758	1.5722	0.0439
Histidine metabolism	15	0.24608	1	0.22071	1.5109	0.24194
Glycerolipid metabolism	18	0.29529	1	0.25887	1.3514	0.0192

Aminoacyl-tRNA biosynthesis	67	1.0991	2	0.30164	1.1985	0
Arachidonic acid metabolism	36	0.59058	1	0.45289	0.7921	0
Biosynthesis of unsaturated fatty acids	42	0.68902	1	0.50598	0.68126	0

Day 21 post-exposure

	Total	Expected	Hits	p-value	-LOG(p)	Impact
**Linoleic acid metabolism**	**5**	**0.089158**	**2**	**0.0029555**	**5.8241**	**1**
**Glycerophospholipid metabolism**	**30**	**0.53495**	**3**	**0.014806**	**4.2127**	**0.275**
**Histidine metabolism**	**15**	**0.26748**	**2**	**0.027817**	**3.5821**	**0.46237**
**Biosynthesis of unsaturated fatty acids**	**42**	**0.74893**	**3**	**0.036358**	**3.3143**	**0**
Sphingolipid metabolism	21	0.37447	2	0.052124	2.9541	0.03008
Phenylalanine, tyrosine and tryptophan biosynthesis	4	0.071327	1	0.069514	2.6662	0
Arachidonic acid metabolism	36	0.64194	2	0.13309	2.0167	0.32601
Taurine and hypotaurine metabolism	8	0.14265	1	0.13438	2.0071	0
alpha-Linolenic acid metabolism	9	0.16049	1	0.1499	1.8978	0
Nitrogen metabolism	9	0.16049	1	0.1499	1.8978	0
Phenylalanine metabolism	9	0.16049	1	0.1499	1.8978	0.24074
Primary bile acid biosynthesis	46	0.82026	2	0.19666	1.6263	0.05952
Glycosylphosphatidylinositol(GPI)-anchor biosynthesis	14	0.24964	1	0.2236	1.4979	0.0439
Fatty acid elongation in mitochondria	27	0.48146	1	0.38762	0.94773	0
Steroid biosynthesis	35	0.62411	1	0.47143	0.75199	0
Fatty acid metabolism	39	0.69544	1	0.50909	0.67514	0
Tryptophan metabolism	41	0.7311	1	0.52694	0.64068	0.01473
Fatty acid biosynthesis	43	0.76676	1	0.54416	0.60851	0
Aminoacyl-tRNA biosynthesis	67	1.1947	1	0.70919	0.34363	0
Steroid hormone biosynthesis	70	1.2482	1	0.72523	0.32126	0

Table S6

Diseases pathway enrichment analysis were carried out using MetaboAnalyst software using human disease-associated metabolite sets which consist of a library contains 344 metabolite sets reported in human blood. Pathway which are significant are bold.

Day 1 post-exposure

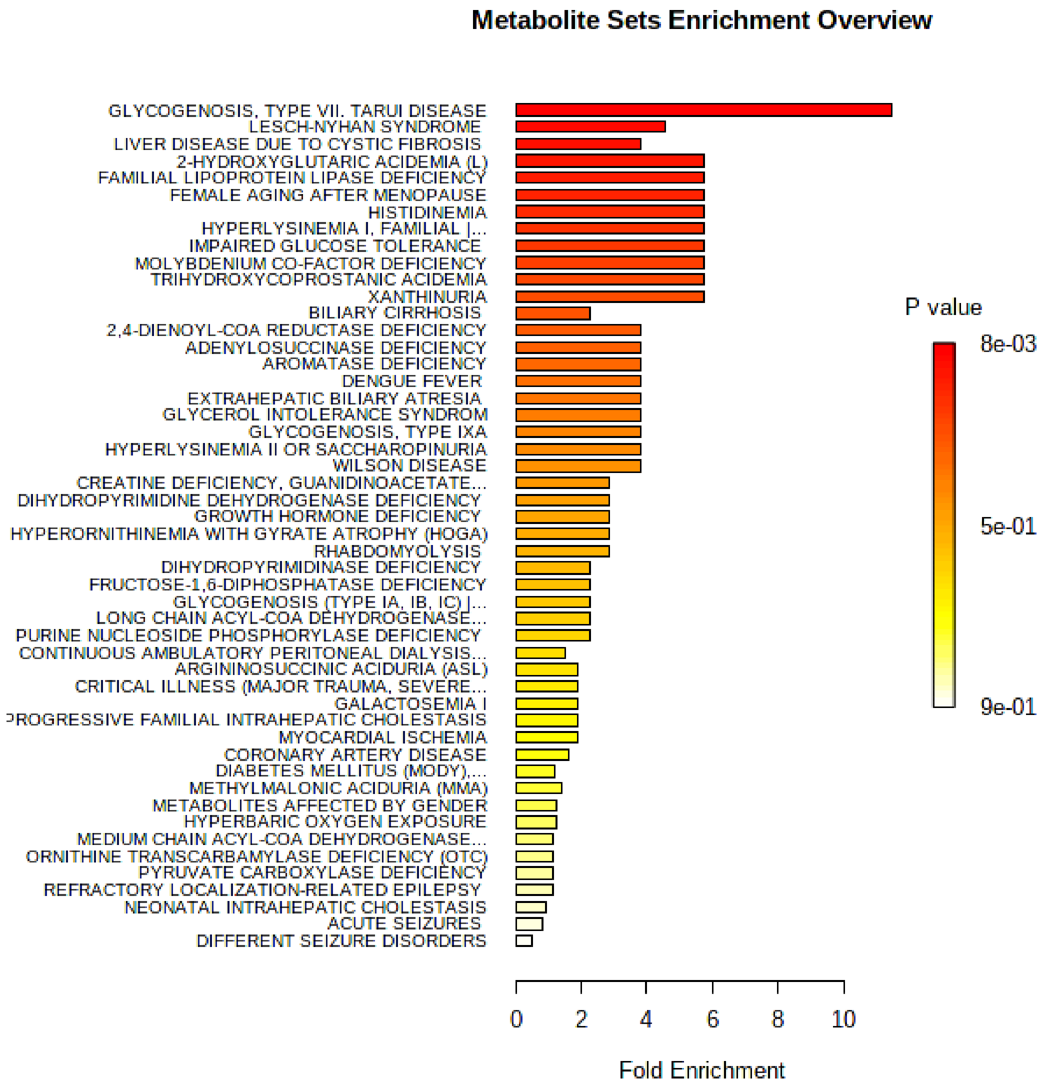

Metabolite Set	Total	Hits	Expect	P value
**GLYCOGENOSIS, TYPE VII. TARUI DISEASE**	**2**	**2**	**0.175**	**0.0075**
LESCH-NYHAN SYNDROME	5	2	0.438	0.0634
LIVER DISEASE DUE TO CYSTIC FIBROSIS	6	2	0.526	0.0898
2-HYDROXYGLUTARIC ACIDEMIA (L)	2	1	0.175	0.168
FAMILIAL LIPOPROTEIN LIPASE DEFICIENCY	2	1	0.175	0.168
FEMALE AGING AFTER MENOPAUSE	2	1	0.175	0.168
HISTIDINEMIA	2	1	0.175	0.168
HYPERLYSINEMIA I, FAMILIAL	HYPERPIPECOLATEMIA	2	1	0.175	0.168
IMPAIRED GLUCOSE TOLERANCE	2	1	0.175	0.168
MOLYBDENIUM CO-FACTOR DEFICIENCY	2	1	0.175	0.168
TRIHYDROXYCOPROSTANIC ACIDEMIA	2	1	0.175	0.168
XANTHINURIA	2	1	0.175	0.168
BILIARY CIRRHOSIS	10	2	0.877	0.216
2,4-DIENOYL-COA REDUCTASE DEFICIENCY	3	1	0.263	0.241
ADENYLOSUCCINASE DEFICIENCY	3	1	0.263	0.241
AROMATASE DEFICIENCY	3	1	0.263	0.241
DENGUE FEVER	3	1	0.263	0.241
EXTRAHEPATIC BILIARY ATRESIA	3	1	0.263	0.241
GLYCEROL INTOLERANCE SYNDROM	3	1	0.263	0.241
GLYCOGENOSIS, TYPE IXA	3	1	0.263	0.241
HYPERLYSINEMIA II OR SACCHAROPINURIA	3	1	0.263	0.241
WILSON DISEASE	3	1	0.263	0.241
CREATINE DEFICIENCY, GUANIDINOACETATE METHYLTRANSFERASE DEFICIENCY	4	1	0.351	0.308
DIHYDROPYRIMIDINE DEHYDROGENASE DEFICIENCY	4	1	0.351	0.308
GROWTH HORMONE DEFICIENCY	4	1	0.351	0.308
HYPERORNITHINEMIA WITH GYRATE ATROPHY (HOGA)	4	1	0.351	0.308
RHABDOMYOLYSIS	4	1	0.351	0.308
DIHYDROPYRIMIDINASE DEFICIENCY	5	1	0.438	0.369
FRUCTOSE-1,6-DIPHOSPHATASE DEFICIENCY	5	1	0.438	0.369
GLYCOGENOSIS (TYPE IA, IB, IC)	GLYCOGENOSIS, TYPE VI. HERS DISEASE	5	1	0.438	0.369
LONG CHAIN ACYL-COA DEHYDROGENASE DEFICIENCY (LCAD)	5	1	0.438	0.369
PURINE NUCLEOSIDE PHOSPHORYLASE DEFICIENCY	5	1	0.438	0.369

CONTINUOUS AMBULATORY PERITONEAL DIALYSIS (CAPD)	15	2	1.32	0.385
ARGININOSUCCINIC ACIDURIA (ASL)	6	1	0.526	0.425
CRITICAL ILLNESS (MAJOR TRAUMA, SEVERE SEPTIC SHOCK, OR CARDIOGENIC SHOCK)	6	1	0.526	0.425
GALACTOSEMIA I	6	1	0.526	0.425
PROGRESSIVE FAMILIAL INTRAHEPATIC CHOLESTASIS	6	1	0.526	0.425
MYOCARDIAL ISCHEMIA	6	1	0.526	0.425
CORONARY ARTERY DISEASE	7	1	0.614	0.476
DIABETES MELLITUS (MODY), NON-INSULIN-DEPENDENT	19	2	1.67	0.51
METHYLMALONIC ACIDURIA (MMA)	8	1	0.701	0.523
METABOLITES AFFECTED BY GENDER	9	1	0.789	0.565
HYPERBARIC OXYGEN EXPOSURE	9	1	0.789	0.565
MEDIUM CHAIN ACYL-COA DEHYDROGENASE DEFICIENCY (MCAD)	10	1	0.877	0.604
ORNITHINE TRANSCARBAMYLASE DEFICIENCY (OTC)	10	1	0.877	0.604
PYRUVATE CARBOXYLASE DEFICIENCY	10	1	0.877	0.604
REFRACTORY LOCALIZATION-RELATED EPILEPSY	10	1	0.877	0.604
NEONATAL INTRAHEPATIC CHOLESTASIS	12	1	1.05	0.672
ACUTE SEIZURES	14	1	1.23	0.728
DIFFERENT SEIZURE DISORDERS	24	1	2.1	0.896

Day 5 post-exposure

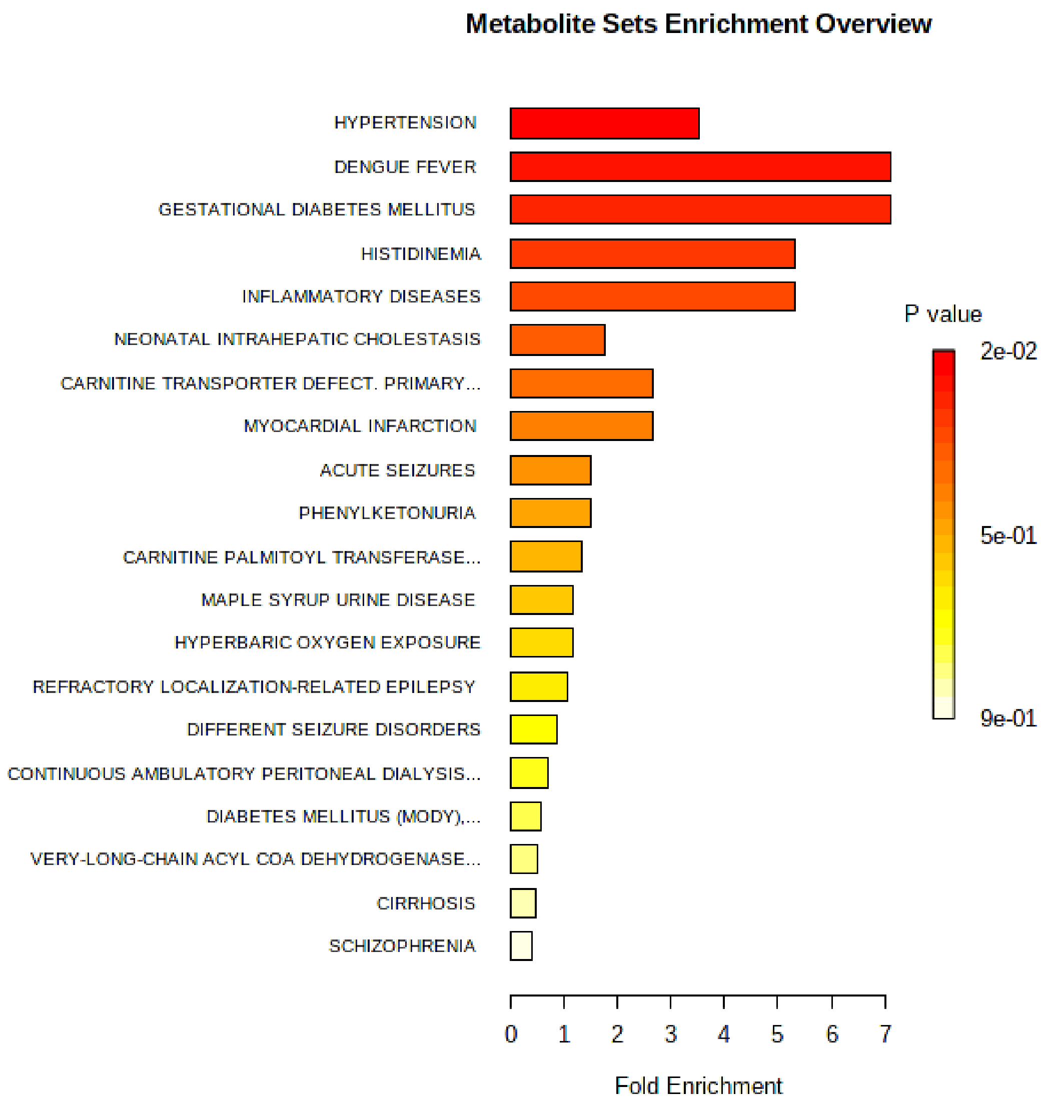

Metabolite Set	Total	Hits	Expect	P value
**HYPERTENSION**	**12**	**4**	**1.13**	**0.0193**
**DENGUE FEVER**	**3**	**2**	**0.282**	**0.0244**
**GESTATIONAL DIABETES MELLITUS**	**3**	**2**	**0.282**	**0.0244**
HISTIDINEMIA	2	1	0.188	0.179
INFLAMMATORY DISEASES	2	1	0.188	0.179
NEONATAL INTRAHEPATIC CHOLESTASIS	12	2	1.13	0.313
CARNITINE TRANSPORTER DEFECT. PRIMARY SYSTEMIC CARNITINE DEFICIENCY	4	1	0.376	0.327
MYOCARDIAL INFARCTION	4	1	0.376	0.327
ACUTE SEIZURES	14	2	1.32	0.385
PHENYLKETONURIA	7	1	0.658	0.501
CARNITINE PALMITOYL TRANSFERASE DEFICIENCY (II)	8	1	0.752	0.549
MAPLE SYRUP URINE DISEASE	9	1	0.846	0.592
HYPERBARIC OXYGEN EXPOSURE	9	1	0.846	0.592
REFRACTORY LOCALIZATION-RELATED EPILEPSY	10	1	0.939	0.631
DIFFERENT SEIZURE DISORDERS	24	2	2.25	0.681
CONTINUOUS AMBULATORY PERITONEAL DIALYSIS (CAPD)	15	1	1.41	0.778
DIABETES MELLITUS (MODY), NON-INSULIN-DEPENDENT	19	1	1.78	0.852
VERY-LONG-CHAIN ACYL COA DEHYDROGENASE DEFICIENCY (VLCAD)	21	1	1.97	0.88
CIRRHOSIS	23	1	2.16	0.902
SCHIZOPHRENIA	26	1	2.44	0.929

Day 9 post-exposure

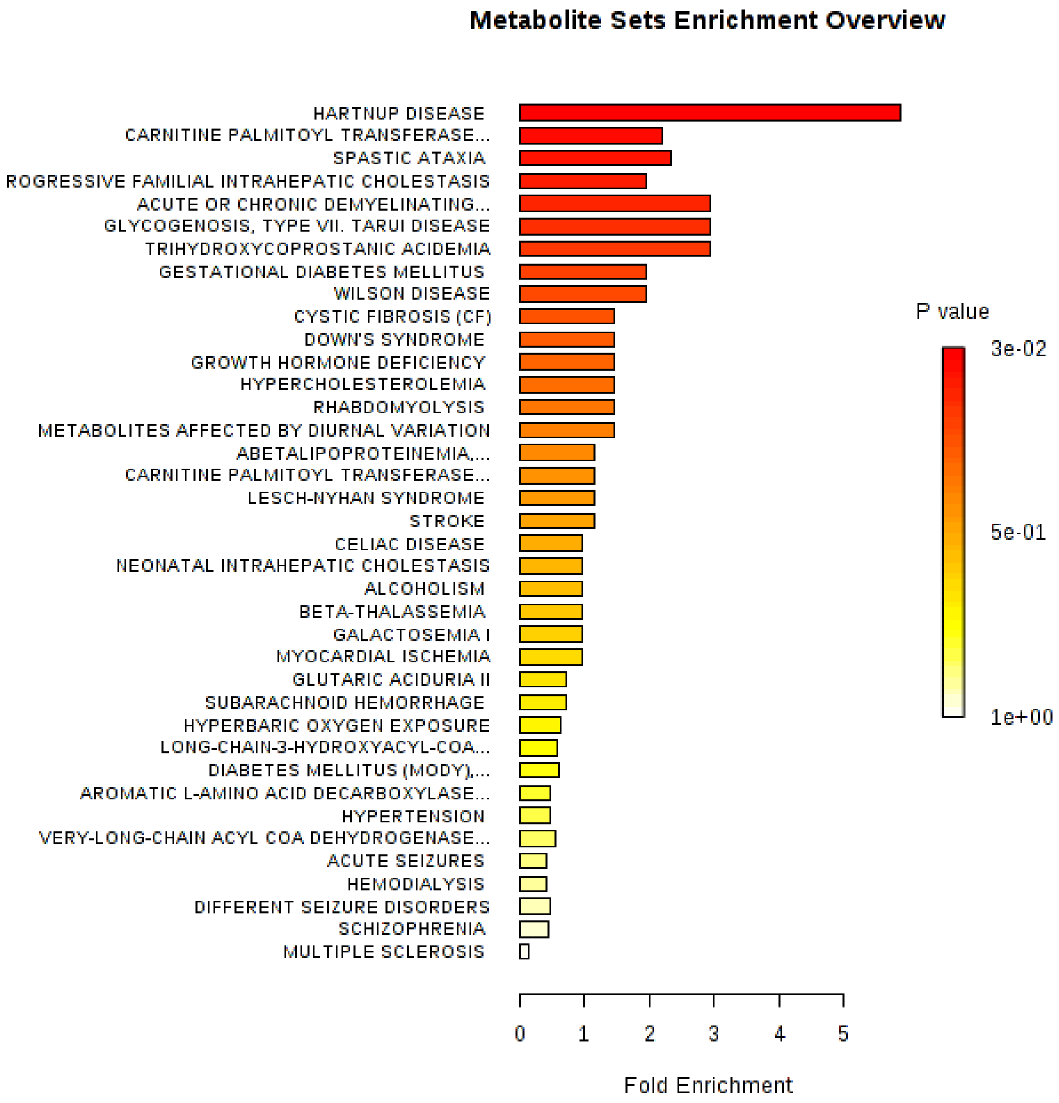

Metabolite Set	Total	Hits	Expect	P value
**HARTNUP DISEASE**	**2**	**2**	**0.342**	**0.029**
CARNITINE PALMITOYL TRANSFERASE DEFICIENCY (II)	8	3	1.37	0.142
SPASTIC ATAXIA	5	2	0.856	0.204
PROGRESSIVE FAMILIAL INTRAHEPATIC CHOLESTASIS	6	2	1.03	0.274
ACUTE OR CHRONIC DEMYELINATING POLYNEUROPATHIES	MILD COGNITIVE IMPAIRMENT	NEUROBORRELIOSIS	2	1	0.342	0.313
GLYCOGENOSIS, TYPE VII. TARUI DISEASE	2	1	0.342	0.313
TRIHYDROXYCOPROSTANIC ACIDEMIA	2	1	0.342	0.313
GESTATIONAL DIABETES MELLITUS	3	1	0.514	0.431
WILSON DISEASE	3	1	0.514	0.431
CYSTIC FIBROSIS (CF)	4	1	0.685	0.529
DOWN'S SYNDROME	4	1	0.685	0.529
GROWTH HORMONE DEFICIENCY	4	1	0.685	0.529
HYPERCHOLESTEROLEMIA	4	1	0.685	0.529
RHABDOMYOLYSIS	4	1	0.685	0.529
METABOLITES AFFECTED BY DIURNAL VARIATION	4	1	0.685	0.529
ABETALIPOPROTEINEMIA, BASSEN-KORNZWEIG-SYNDROME, ACANTHOCYTOSIS (ABL)	5	1	0.856	0.611
CARNITINE PALMITOYL TRANSFERASE DEFICIENCY (I)	5	1	0.856	0.611
LESCH-NYHAN SYNDROME	5	1	0.856	0.611
STROKE	5	1	0.856	0.611
CELIAC DISEASE	12	2	2.05	0.638
NEONATAL INTRAHEPATIC CHOLESTASIS	12	2	2.05	0.638
ALCOHOLISM	6	1	1.03	0.678
BETA-THALASSEMIA	6	1	1.03	0.678
GALACTOSEMIA I	6	1	1.03	0.678
MYOCARDIAL ISCHEMIA	6	1	1.03	0.678
GLUTARIC ACIDURIA II	8	1	1.37	0.78
SUBARACHNOID HEMORRHAGE	8	1	1.37	0.78
HYPERBARIC OXYGEN EXPOSURE	9	1	1.54	0.818
LONG-CHAIN-3-HYDROXYACYL-COA DEHYDROGENASE DEFICIENCY (LCHAD)	10	1	1.71	0.85
DIABETES MELLITUS (MODY), NON-INSULIN-DEPENDENT	19	2	3.25	0.866
AROMATIC L-AMINO ACID DECARBOXYLASE DEFICIENCY	12	1	2.05	0.898
HYPERTENSION	12	1	2.05	0.898

VERY-LONG-CHAIN ACYL COA DEHYDROGENASE DEFICIENCY (VLCAD)	21	2	3.59	0.902
ACUTE SEIZURES	14	1	2.4	0.931
HEMODIALYSIS	14	1	2.4	0.931
DIFFERENT SEIZURE DISORDERS	24	2	4.11	0.939
SCHIZOPHRENIA	26	2	4.45	0.956
MULTIPLE SCLEROSIS	39	1	6.68	1

Day 13 post-exposure

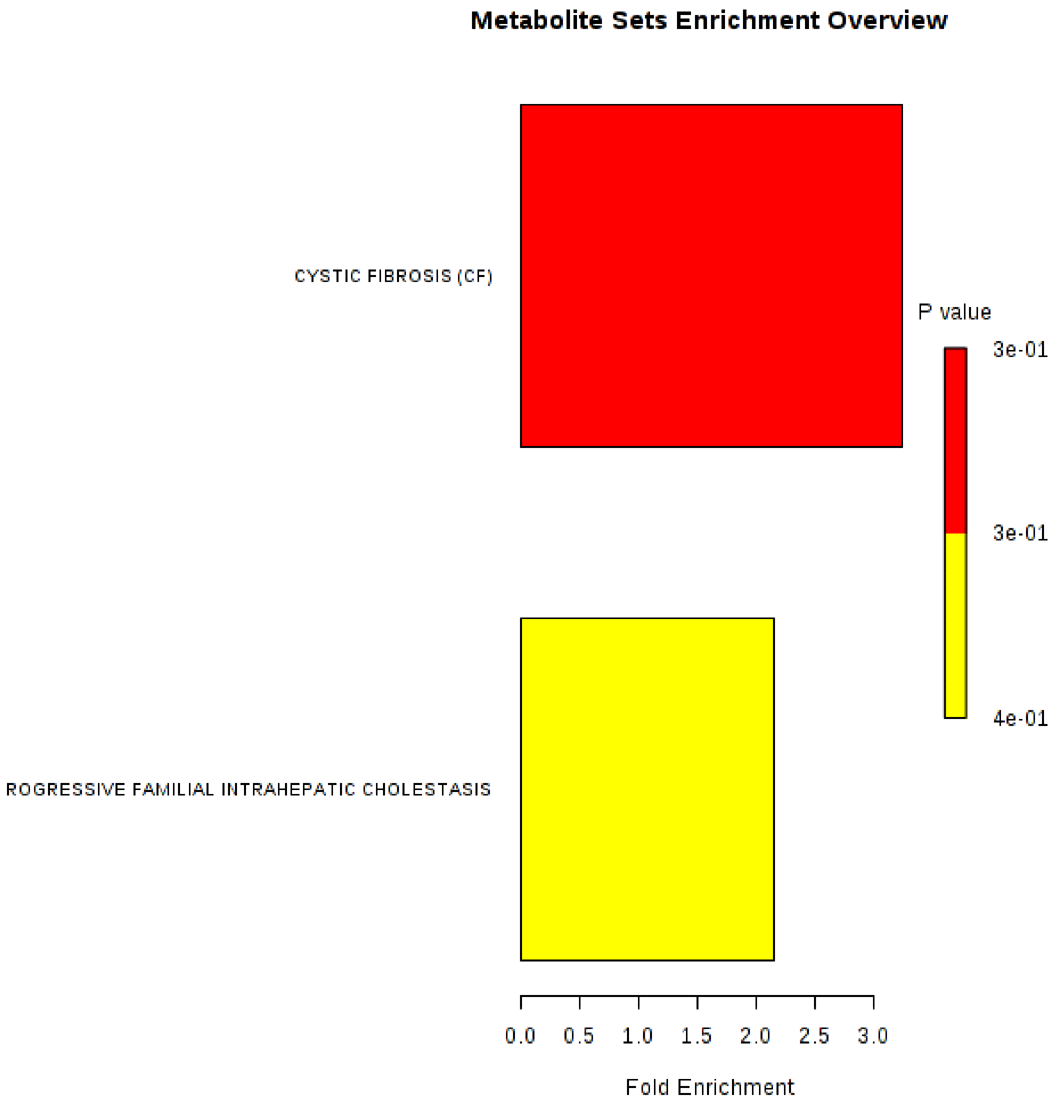

	Metabolite Set	Total	Hits	Expect	P value
	CYSTIC FIBROSIS (CF)	4	1	0.309	0.276
	PROGRESSIVE FAMILIAL INTRAHEPATIC CHOLESTASIS	6	1	0.463	0.384

Day 17 post-exposure

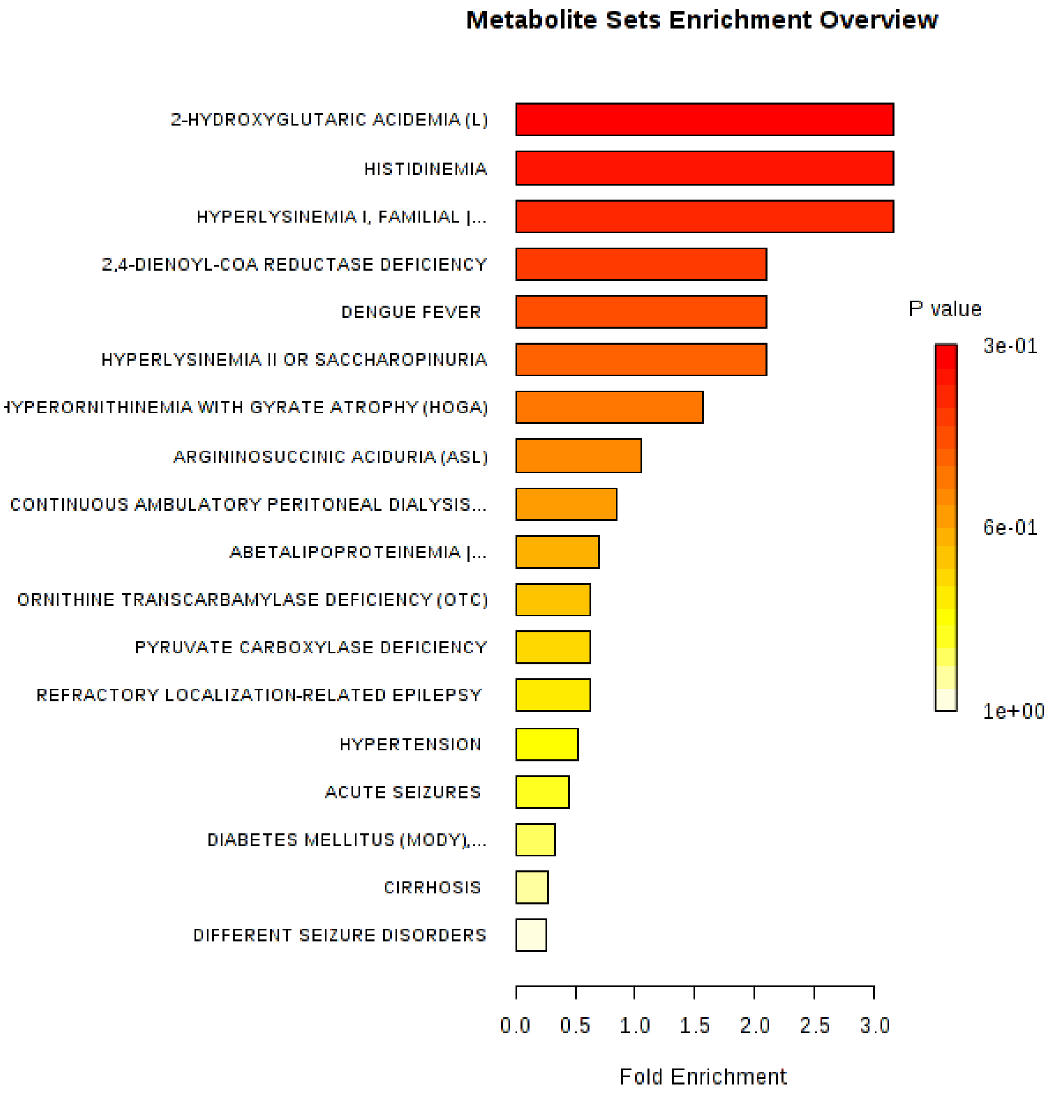

	Metabolite Set	Total	Hits	Expect	P value
	2-HYDROXYGLUTARIC ACIDEMIA (L)	2	1	0.317	0.292
	HISTIDINEMIA	2	1	0.317	0.292
	HYPERLYSINEMIA I, FAMILIAL | HYPERPIPECOLATEMIA	2	1	0.317	0.292
	2,4-DIENOYL-COA REDUCTASE DEFICIENCY	3	1	0.476	0.405
	DENGUE FEVER	3	1	0.476	0.405
	HYPERLYSINEMIA II OR SACCHAROPINURIA	3	1	0.476	0.405
	HYPERORNITHINEMIA WITH GYRATE ATROPHY (HOGA)	4	1	0.635	0.5
	ARGININOSUCCINIC ACIDURIA (ASL)	6	1	0.952	0.647
	CONTINUOUS AMBULATORY PERITONEAL DIALYSIS (CAPD)	15	2	2.38	0.718
	ABETALIPOPROTEINEMIA | HYPOBETALIPOPROTEINEMIA	9	1	1.43	0.792
	ORNITHINE TRANSCARBAMYLASE DEFICIENCY (OTC)	10	1	1.59	0.825
	PYRUVATE CARBOXYLASE DEFICIENCY	10	1	1.59	0.825
	REFRACTORY LOCALIZATION-RELATED EPILEPSY	10	1	1.59	0.825
	HYPERTENSION	12	1	1.9	0.877
	ACUTE SEIZURES	14	1	2.22	0.914
	DIABETES MELLITUS (MODY), NON-INSULIN-DEPENDENT	19	1	3.01	0.965
	CIRRHOSIS	23	1	3.65	0.983
	DIFFERENT SEIZURE DISORDERS	24	1	3.81	0.986

Day 21 post-exposure

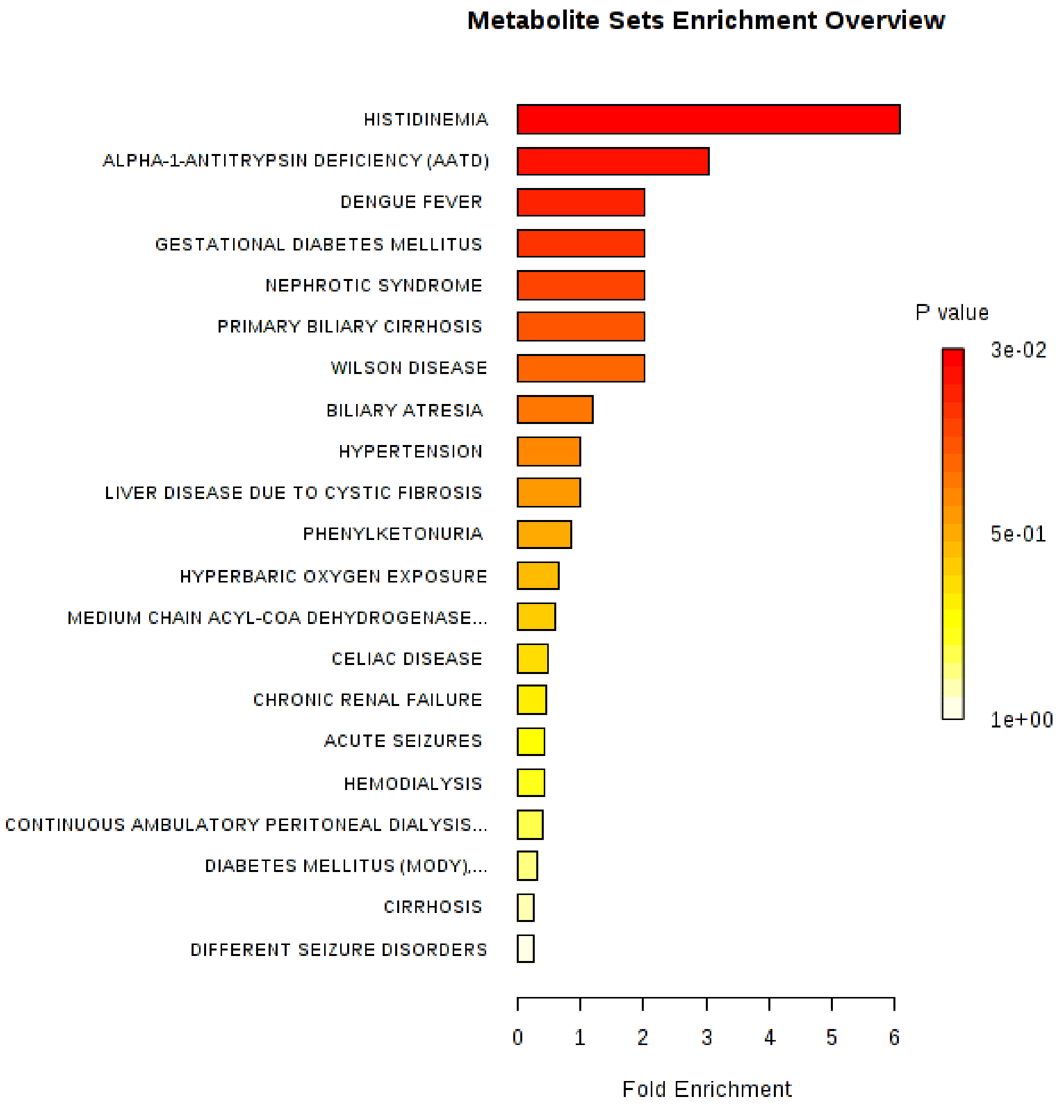

Metabolite Set	Total	Hits	Expect	P value
**HISTIDINEMIA**	**2**	**2**	**0.33**	**0.0269**
ALPHA-1-ANTITRYPSIN DEFICIENCY (AATD)	2	1	0.33	0.303
DENGUE FEVER	3	1	0.495	0.418
GESTATIONAL DIABETES MELLITUS	3	1	0.495	0.418
NEPHROTIC SYNDROME	3	1	0.495	0.418
PRIMARY BILIARY CIRRHOSIS	3	1	0.495	0.418
WILSON DISEASE	3	1	0.495	0.418
BILIARY ATRESIA	5	1	0.825	0.596
HYPERTENSION	12	2	1.98	0.616
LIVER DISEASE DUE TO CYSTIC FIBROSIS	6	1	0.99	0.663
PHENYLKETONURIA	7	1	1.15	0.719
HYPERBARIC OXYGEN EXPOSURE	9	1	1.48	0.805
MEDIUM CHAIN ACYL-COA DEHYDROGENASE DEFICIENCY (MCAD)	10	1	1.65	0.838
CELIAC DISEASE	12	1	1.98	0.888
CHRONIC RENAL FAILURE	13	1	2.14	0.907
ACUTE SEIZURES	14	1	2.31	0.923
HEMODIALYSIS	14	1	2.31	0.923
CONTINUOUS AMBULATORY PERITONEAL DIALYSIS (CAPD)	15	1	2.47	0.936
DIABETES MELLITUS (MODY), NON-INSULIN-DEPENDENT	19	1	3.13	0.97
CIRRHOSIS	23	1	3.79	0.986
DIFFERENT SEIZURE DISORDERS	24	1	3.96	0.988

References

1. Dunn, W. B.; Broadhurst, D.; Begley, P.; Zelena, E.; Francis-McIntyre, S.; Anderson, N.; Brown, M.; Knowles, J. D.; Halsall, A.; Haselden, J. N.; Nicholls, A. W.; Wilson, I. D.; Kell, D. B.; Goodacre, R.; Human Serum Metabolome, C., Procedures for large-scale metabolic profiling of serum and plasma using gas chromatography and liquid chromatography coupled to mass spectrometry. *Nat Protoc* **2011,** *6* (7), 1060-83.
2. Benjamini, Y.; Krieger, A. M.; Yekutieli, D., Adaptive linear step-up procedures that control the false discovery rate. *Biometrika* **2006,** *93* (3), 491-507.
3. Law, K.; Han, T.-L., *The Good, the Better and the Best of Progenesis QI*. 2016.
4. Wishart, D. S.; Xia, J., MetPA: a web-based metabolomics tool for pathway analysis and visualization. *Bioinformatics* **2010,** *26* (18), 2342-2344.
